# Multiscale design of stiffening and ROS scavenging hydrogels for the augmentation of mandibular bone regeneration

**DOI:** 10.1016/j.bioactmat.2022.05.021

**Published:** 2022-05-23

**Authors:** Yanlin Wu, Xuan Li, Yimin Sun, Xiujun Tan, Chenglin Wang, Zhenming Wang, Ling Ye

**Affiliations:** State Key Laboratory of Oral Diseases, West China Hospital of Stomatology, Sichuan University, Chengdu, 610041, China

**Keywords:** Biomimetic hydrogels, Multiscale design, ROS scavenging, Stiffness, Bone regeneration

## Abstract

Although biomimetic hydrogels play an essential role in guiding bone remodeling, reconstructing large bone defects is still a significant challenge since bioinspired gels often lack osteoconductive capacity, robust mechanical properties and suitable antioxidant ability for bone regeneration. To address these challenges, we first engineered molecular design of hydrogels (gelatin/polyethylene glycol diacrylate/2-(dimethylamino)ethyl methacrylate, GPEGD), where their mechanical properties were significantly enhanced via introducing trace amounts of additives (0.5 wt%). The novel hybrid hydrogels show high compressive strength (>700 kPa), stiff modulus (>170 kPa) and strong ROS-scavenging ability. Furthermore, to endow the GPEGD hydrogels excellent osteoinductions, novel biocompatible, antioxidant and BMP-2 loaded polydopamine/heparin nanoparticles (BPDAH) were developed for functionalization of the GPEGD gels (BPDAH-GPEGD). *In vitro* results indicate that the antioxidant BPDAH-GPEGD is able to deplete elevated ROS levels to protect cells viability against ROS damage. More importantly, the BPDAH-GPEGD hydrogels have good biocompatibility and promote the osteo differentiation of preosteoblasts and bone regenerations. At 4 and 8 weeks after implantation of the hydrogels in a mandibular bone defect, Micro-computed tomography and histology results show greater bone volume and enhancements in the quality and rate of bone regeneration in the BPDAH-GPEGD hydrogels. Thus, the multiscale design of stiffening and ROS scavenging hydrogels could serve as a promising material for bone regeneration applications.

## Highlights

• Trace additives of DMAEMA markedly enhanced the mechanical performances of the gelatin-based hydrogels through molecular induced multiple crosslinking structures.
• Molecular design strategy combined with bioactive nanocomposites have a synergistically effects on promoting ROS scavenging ability and osteoactivity of the biomimetic hydrogels.

## Graphical abstract

**Figure:**
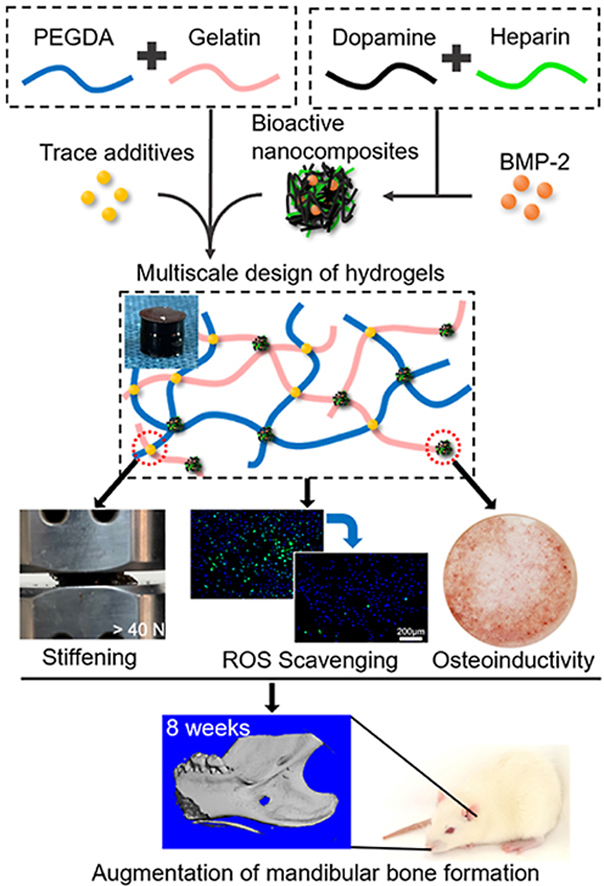

## 1 Introduction

Oral and maxillofacial bone plays a critical role in mastication, speech, swallowing and facial profile aesthetics. Specifically, mandibular bone defects caused by trauma, tumor resection, infection periodontitis, and congenital abnormalities can impair quality of life. Small-sized bone defects generally exhibit self-healing ability, critical-sized facial bone defects, however, remain a major challenge to clinicians in term of functional and aesthetic restoration. The traditional autologous and allogenic bone grafts is the gold standard for the repair of large bone defects. Unfortunately, shortage of sources, donor site morbidity, anatomical shapes limit their applications. Thus, engineered biomaterials, enabling the recapitulation of local bone microenvironments to direct the fate of endogenous cells by optimized biophysical and biochemical cues of the implants, have recently emerged as an alternative in promoting new bone formation [1,2]. Since bone remodeling is a complex procedure, a range of intrinsic characteristic are required for an ideal bone substitute, such as suitable mechanical properties, suitable biodegradable rate, excellent biocompatibility, spatiotemporal regulation of bioactive cues [3].

Recent studies report gelatin-based hydrogels can imitate the extracellular matrix (ECM) of bone tissue with versatile functions due to their good biocompatibility, enzyme degradation ability, and multiple cell-binding domains behavior [4]. However, the application of gelatin hydrogels in bone repair is severely hampered owing to their low toughness and limited mechanical stiffness. Therefore, much efforts have been devoted to engineering mechanically reinforced gelatin hydrogels by using various physically or covalently crosslinked strategies including slidable supramolecular cross-linkers [5], hofmeister effects [[6], [7], [8]], ion-induced reversible crosslinking by hybridized polymers [9], double network structures [10], conjoined-networks [11], nanocomposite crosslinked additives [12,13]. The exhibiting single reversible approach, however, often results in quickly degrading hydrogels even in few days while the covalent crosslinked method might lead to brittle properties, which was not suitable for improving bone tissue reconstruction. Therefore, how to construct a mechanical reinforced gelatin-based hydrogels with suitable degradable ratio still remains a challenge.

Moreover, large bone defects are often accompanied with elevated levels of reactive oxygen species (ROS) during bone healing, resulting in oxidative stress, which could induce apoptosis and necrosis of osteoblasts and osteocytes, inhibit mesenchymal cell osteogenic differentiation, and thereby, delay bone reconstruction [14,15]. To this end, tremendous efforts have been made to construct advanced bone implants with ROS scavenging ability through functionalization of various types of antioxidants such as biopolymers [16,17], polyphenol nanostructures [18], inorganic structures [19,20], composite coatings [21], metal ions [22] or small molecules [23,24] for depletion of excess ROS and thereby promoting bone tissue regeneration. Among them, biogenic gelatin polymers and mussel-inspired polydopamine (PDA) nanoparticles show strong ROS scavenging capacity and superior biocompatibility [[25], [26], [27], [28]]. Hence, decoration of gelatin-based biomaterials with PDA nanoparticles for the depletion of excess ROS at large bone defect sites may offer effective approach to promote bone repair.

On the other hand, the procedure of bone remodeling is also spatiotemporally mediated by a series of growth factors. As an important osteogenesis promoting factor, bone morphogenetic protein-2 (BMP-2) has been widely used for repair large bone fractures in clinical applications [[29], [30], [31]]. More importantly, extensive studies have reported that BMP-2 tethered hydrogels could stimulate cellular pathways responsible for osteogenesis and enhance bone formation in animal experiments [[32], [33], [34]]. Given the short half-life and rapid clearance of the BMP-2 from the body, traditionally covalent or weak electrostatic encapsulation of BMP-2 into natural hydrogels might decrease the bioactivity or somehow trigger the occurrence of side effects *in vivo* [35,36]. Heparin, a naturally occurring glycosaminoglycan, is highly negatively charged and has a strong affinity for a class of positively charged growth factors such as vascular endothelial growth factor (VEGF), fibroblast growth factors (FGFs), insulin-like growth factors (IGFs), and bone morphogenetic proteins (BMPs) [[37], [38], [39], [40], [41]]. Notably, heparin with strong affinity to BMP-2 molecules has been incorporated into many biomaterials such as hydrogels, microparticles, nanoparticles, and coatings to prolong BMP-2 release *in vitro* and *in vivo*, resulting in amplifying BMP-2 bioactivity [[42], [43], [44], [45]]. Inspired by the peculiar nature of ECM in which BMP-2 delivery and binding can be more precisely controlled by the sulfated glycosaminoglycans (GAGs) heparin via strong affinity interactions, heparin-functionalized bone implants have been particularly widely used for BMP-2 immobilization to stabilize the molecule against denaturation or proteolysis and enhances their osteoactivity.

To meet the multiple requirements of the bone grafts, a stiffening and bioactive gelatin-based hydrogel was constructed through multiscale strategy for ROS scavenging, precisely controlled release of BMP-2, and thereby promoting critical bone formation. Initially, ROS scavenging PDA/heparin (PDAH) nanoparticles were prepared to improve the encapsulation efficiency and control BMP-2 release behavior. Simultaneously, molecular design of stiffening gelatin/polyethylene glycol diacrylate/2-(dimethylamino)ethyl methacrylate (GPEGD) hydrogel crosslinked by both hydrogen bonds and covalent bonds were subtly synthesized. To endow the gelatin-based hydrogels with high osteoinduction and robust ROS scavenging ability, BMP-2 incorporated PDAH nanoparticles were introduced to the optimized GPEGD hydrogels to obtain nanocomposite hydrogels. We hypothesize that the proposed nanocomposite hydrogels with suitable mechanical properties, biodegradation ability, strong ROS scavenging activity, and sustain release of BMP-2 would have strong potential in mandibular bone tissue applications.

## 2 Materials and methods

### 2.1 Materials

Gelatin (porcine skin, type A), dopamine hydrochloride, 2-(dimethylamino)ethyl methacrylate (DMAEMA), polyethylene glycol diacrylate (PEGDA), visible light initiator lithium phenyl(2,4,6-trimethylbenzoyl) phosphinate (LAP), DCFH-DA, Hoechst 33342, β-sodium glycerophosphate, l-ascorbic acid and dexamethasone were purchased from Sigma Aldrich (St. Louis, MO, USA). Heparin sodium salt (Mn = 1.25 kDa, >189 U/mg, Sinopharm Chemical Reagent Co. Shanghai, China), 2, 2-diphenyl-1-picrylhydrazyl (DPPH) (Mackline, China), Recombinant Human/Murine/Rat BMP-2 (E.coli derived, PeproTech, USA), Human/Murine/Rat BMP-2 Standard TMB ELISA Development Kit (PeproTech, USA), Live/Dead Staining Kit (Beyotime, China), CCK8 assay (ApexBio, USA), fetal bovine serum (FBS; HyClone, USA), α-MEM (HyClone, USA), 1% penicillin-streptomycin solution (HyClone, USA), trypsin (Gibco, USA), type II collagenase (BioFroxx, Germany), DAPI (Abcam, USA), Alizarin Red S (ARS) Staining Kit (Beyotime, China), Alp staining (Beyotime, China), Hematoxylin and Eosin (H&E) Staining Kit (Solarbio, China) and Masson's Trichrome Stain Kit (Solarbio, China) were used for *in vitro* and *in vivo* study. Unless otherwise stated, all other regents and solvents were used as received without further purification or modification.

### 2.2 Synthesis and characteristic of polydopamine (PDA), PDA/heparin (PDAH) and BMP-2 loaded-PDAH (BPDAH) nanoparticles

PDA and PDAH nanoparticles were synthesized through an oxidation and self-polymerization method according to the modified procedures described in our previous publications [46,47]. Briefly, ammonia aqueous solution (0.8 mL, 28–30%), ethanol (40 mL) and deionized water (90 mL) were mixed under mild stirring at room temperature for 10 min. Dopamine hydrochloride (0.5 g) without or with different weights of heparin (0.1, 0.25 and 0.5 g) were dissolved in deionized water (10 mL) and then injected into the above mixture solution. Next, the mixture was stirred to allow dopamine polymerization at room temperature in the dark for 30 h. The formed nanoparticle solutions were centrifuged (12000 g, 10 min) and purified by with ethanol. Note that the nanoparticles prepared by using different chemical compositions of PDA (0.5 g), PDA (0.5 g)/heparin (0.1 g), PDA (0.5 g)/heparin (0.25 g) and PDA (0.5 g)/heparin (0.5 g) in the deionized waters were denoted as PDA, PDAH1, PDAH2 and PDAH3, respectively. As for preparation of BPDAH nanoparticles, BMP-2 (1 μg) was incubated with PDAH1 (1 mL, 1 mg mL^−1^) under stirring conditions for 24 h followed by purification and centrifugation. BMP-2 ELISA Kits were used to detect the BMP-2 concentrations in the supernatants to obtain BMP-2 encapsulation ratio in different nanoparticles. In our study, the PDAH1, not otherwise specified, refers to PDAH nanoparticles. The morphology and elemental compositions of nanoparticles were examined by scanning electron microscopy (SEM) with X-ray microanalysis (JSM 6390, JEOL, Japan). The diameters of different types of particles were analyzed by Image J software. The chemical functions of the particles were characterized using a Fourier transform infrared spectrophotometry (FT-IR) system (Model 5700, Nicolet, Germany).

### 2.3 *In vitro* reactive oxygen species (ROS) scavenging activities of PDA and PDAH

The ROS scavenging properties of PDA and PDAH were investigated by determining the scavenging efficiency on DPPH radicals. 100 μL of PDA and PDAH solution (5, 10, 25 and 50 μg mL^−1^) were prepared in a 48-well plate. Then, 100 μL of DPPH solution (0.1 mM in ethanol) was added and incubated for 60 min in dark. As the blank group, 100 μL of ethanol was used instead of DPPH solution. As the control group, 100 μL of deionized water was used instead of PDA/PDAH solution. After incubation, the resulting mixtures were centrifuged (12000 g, 10 min), and the supernatants were transferred to a 96-well plate. The absorbance (A) at 517 nm of the mixture reaction was measured, and the scavenging efficiency of PDA and PDAH on DPPH radicals was calculated using the following equation:

$$Scavenge\;efficiency\;\%=\left(1-\frac{A_{sample}-A_{blank}}{A_{control}}\right)\times 100\%$$

where A_sample_ represents the absorbance of the PDA or PDAH group, A_blank_ is the absorbance of blank group and A_control_ is the absorbance of control group.

### 2.4 Fabrication of stiffening and nanocomposite hydrogels

Engineered stiffening and nanocomposite hydrogels with different crosslinked polymer networks were synthesized by a one-pot and in-situ free radical polymerization approach. In detail, desired amounts of PEGDA, DMAEMA and BPDAH nanoparticles were added into pure PEGDA aqueous solutions at 37 °C with different weights for the fabrication of various hydrogels as summarized in Supplementary Table S1. The mixtures were stirred until a homogenized solution were formed. The LAP (0.4 wt %) were subsequently added to the mixtures and stirred for 15 min. Next, the mixtures were deoxygenized and then transferred into poly (tetrafluoroethylene) (PTFE) molds. The PEG, GPEG, GPEGD and BPDAH-GPEGD/BMP-GPEGD hydrogels were cured after exposed to UV light (6.9 W cm^−2^, wavelength, 360–480 nm) for 2 min at room temperature. Unless otherwise noted, the GPEGD5 was named as GPEGD.

### 2.5 Mechanical properties and microstructures analysis of various hydrogels

The compression properties of the fresh PEG hydrogel, GPEG hydrogel, GPEGD hydrogel and BPDAH-GPEGD hydrogel were investigated with a universal machine (Instron model 5567) in air at room temperature. The cylindrical samples (n = 6) with a height of 8 mm and a diameter of 6 mm were used for compression tests at a speed of 1 mm/min. The moduli were determined from the slopes in the initial elastic portion (0.1–0.2 strain) of the stress-strain profiles. Various hydrogels were imaged at low vacuum using SEM instruments to observe the microstructures.

### 2.6 Degradation and BMP-2 release and swelling of hydrogels

To evaluate the degradable properties, the initial state of the hydrogel was lyophilized and weighed as W_0_. Then the lyophilized gels with a height of 5 mm and a diameter of 6 mm were immersed in 5 mL of PBS without and within type II collagenase (1 mg mL^−1^) at 70 rpm at 37 °C. At the predetermined time, the medium was exchanged with fresh buffer, and the hydrogels were taken out followed by lyophilized, and weighted again (W_1_). The degradation ratio was calculated as follows:

$$Weight\;remainging\;ratio\;\left(\%\right)=\frac{W_{0}-W_{1}}{W_{0}}\times 100\%$$

The amount of total BMP-2 released in the PBS solution was quantified by BMP-2 Elisa Kits to evaluate the BMP-2 release performance from BPDAH-GPEGD and BMP-GPEGD hydrogels.

To determine the swelling ratio and water uptake ability of the hydrogels, the swelling ratio was calculated by the equitation according to previous studies [48,49]:

$$Swelling\;ratio\;\left(\%\right)=\;\frac{W_{s}-W_{d}}{W_{d}}\times 100\%$$

where Ws represents the mass of gel in swollen state at equilibrium and W_d_ is the mass of freeze-dried hydrogel at its initial state.

### 2.7 Cell compatibility and proliferation evaluations

The pre-osteoblast cell lines (MC3T3-E1, ATCC, USA) cells were used for *in vitro* assay. The MC3T3 were cultured in α-MEM supplemented with 10% FBS, 1% penicillin-streptomycin in a 5% CO^2^ incubator at 37 °C and the medium was replaced every 3 days. To test the compatibility and adhesion behaviors of the hydrogels, various hydrogels (50 μL volume) were prepared in 48-well plates, and then MC3T3 were trypsinized and seeded onto the hydrogels at a density of 2 × 10^4^ cells/hydrogel in normal growth media. After 1-day incubation, the cells were gently rinsed with PBS and then stained with a Live/Dead Staining Kit according to the manufacture's protocol. The stained cells were imaged using an epifluorescence microscope (Leica, Germany). Next, cell proliferation of various hydrogels MC3T3 were quantitatively measured using CCK8 assay at designed time points.

### 2.8 *In vitro* reactive oxygen species (ROS) scavenging activities of hydrogels

#### 2.8.1 The DPPH scavenging efficiency of different hydrogels

The ROS scavenging properties of various hydrogels were investigated by determining the scavenging efficiency on DPPH radicals. 500 μL of different hydrogels were prepared in a 24-well plate. Then, 500 μL of DPPH solution (0.1, 0.5 and 1 mM in ethanol) was added and incubated with hydrogel for 60 min in dark condition. As for the blank group, 500 μL of ethanol was used instead of DPPH solution. DPPH solution was selected as the control group. After incubation, the resulting mixtures were centrifuged (12000 g, 10 min), and the supernatants were transferred to a 96-well plate. The absorbance (A) at 517 nm of the mixture reaction was measured, and the scavenging efficiency of different hydrogels on DPPH radicals was calculated using the following equation:

$$Scavenge\;efficiency\;\%=\left(1-\frac{A_{sample}-A_{blank}}{A_{control}}\right)\times 100\%$$

where A_sample_ represents the absorbance of the hydrogel group, A_blank_ represents the absorbance of blank group and A_control_ represents the absorbance of control group.

#### 2.8.2 Antioxidant properties of hydrogels

Cell protective effect of hydrogels under ROS microenvironment were conducted to evaluate the antioxidant properties of the prepared hydrogels. The cell protective effect of all the hydrogels against oxidative stress were evaluated by CCK-8 assay and Live/Dead Staining Kit. The PEG, GPEG, GPEGD and BPDAH-GPEGD hydrogels (2 mL volume) were treated in 10 mL medium (α-MEM, 10% FBS, 1% penicillin-streptomycin) at 37 °C for 24 h to obtain the extract medium. MC3T3 cells were seeded in a 48-well tissue culture plate (2 × 10^4^ cells/well) and incubated under a 5% CO_2_ atmosphere at 37 °C for 24 h. Then, the medium was replaced with the extract media containing 400 μM or 800 μM H_2_O_2_. After 10 h of incubation, the media was aspirated, and the cells were washed carefully with PBS for 2 times. Next, CCK-8 assay and Live/Dead Staining Kit were used to evaluate the cell viability treated with different hydrogels.

#### 2.8.3 Measurement of intracellular ROS

The effect of various hydrogels on intracellular ROS production in MC3T3 cells under oxidative stress was evaluated using 2′,7′-dichlorodihydrofluorescein diacetate (DCFH-DA) assay. The extract medium was prepared and cells were cultured as described above. The medium was then replaced with the extract media containing 400 μM or 800 μM H_2_O_2_ and the cells were incubated for 1 h. Next, the media was aspirated, and the cells were washed carefully with PBS for 2 times. The cells were co-stained with 10 μM DCFH-DA solution and Hoechst 33342 at 37 °C for 1 h in dark conditions. The fluorescence intensity of DCFH-DA was investigated using a microplate reader (Ex. 488 nm; Em. 525 nm) and observed using an epifluorescence microscope.

### 2.9 *In vitro* osteogenesis study

To assess *in vitro* osteogenic differentiation of MC3T3 cells on the GPEGD, BPDAH-GPEGD and BMP-GPEGD hydrogels, the osteogenic activity and calcium content of cells were measured through alkaline phosphatase (ALP) and Alizarin Red S (ARS) staining. Similarly, various hydrogels (100 μL volume) were prepared in 24-well plates, and then MC3T3 were trypsinized and seeded onto the hydrogels at a density of 5 × 10^4^ cells/hydrogel cultured in induction medium (α-MEM, 5% FBS, 1% penicillin-streptomycin, 10^−2^ M β-sodium glycerophosphate, 50 μg mL^−1^ l-ascorbic acid and 10^−7^ M dexamethasone). After 4 and 7 days of culture, the cells were fixed in 4% paraformaldehyde, followed by a reaction with ALP staining solution for 30 min. After 14 days of culture, the cells were stained with Alizarin Red S (ARS) Staining Kit for 10 min. Then, samples were washed with PBS and imaged using an epifluorescence microscope. Cells cultured for 0 day was denoted as control group.

### 2.10 Gene expression

MC3T3 were seeded in a 6-well plate at a density of 2 × 10^5^ cells per well with osteogenic differentiation media overnight. Then, the hydrogels (500 μL volume) were immersed in the media, and the cells were cultured with the hydrogels for 4, 7 and 14 days. The media was changed every 3 days. The expression of various osteogenic genes, including ALP, bone sialoprotein (BSP), osteocalcin (OCN) and osterix (OSX) were analyzed by the RT-qPCR. Glyceraldehyde phosphate dehydrogenase (GAPDH) was used as the internal control. Cells cultured for 0 day were setted as control. The RT-qPCR was performed with the following forward and reverse primers: ALP: GACAAGAAGCCCTTCACAGC, CTGGGCCTGGTAGTTGTTGT; BSP: ATGGAGACGGCGATAGTTCC, CTAGCTGTTACACCCGAGAGT; OCN: GAACAGACAAGTCCCACACAGC, TCAGCAGAGTGAGCAGAAAGAT; OSX: GGAAAGGAGGCACAAAGAAGC, CCCCTTAGGCACTAGGAG; GAPDH: ACCCAGAAGACTGAGGATGG, TTCAGCTCAGGGATGACCTT.

### 2.11 Animal experiments

All the animal surgical procedures were approved by the Animal Use and Care Committee of Sichuan University (WCHSIRB-D-2020-441). Sprague-Dawley rats (Male, 8 weeks old, body weight: 300–350 g) were used for experiments.

#### 2.11.1 Subcutaneous transplantation experiment

The rats were anesthetized by intraperitoneal injection of pentobarbital, GPEGD hydrogels and BPDAH-GPEGD hydrogels were subcutaneously transplanted into the backs of recipient animals. The soft tissues were repositioned and sutured with silk sutures to achieve primary closure. After 2 weeks post-surgery, the rats were sacrificed, the hydrogels and surrounding soft tissue were harvested and paraffin embedded. Histomorphological analysis was performed on 5 μm thick sections, and the sections were stained with Hematoxylin and Eosin (H&E) staining, and images were taken with a microscope (Leica, Germany).

#### 2.11.2 Establishment and implantation of mandibular bone defect model

The rats were anesthetized by intraperitoneal injection of pentobarbital, and then a full-thickness 5 mm-diameter circle defects were made in mandibular ramus according to previous study [50]. As shown in Fig. S6, the defect circle is located 1 mm from the edges of the mandible. The cylinder hydrogel (n = 5 for each group, 5 mm diameter and 0.5 mm thickness) was implanted into the mandibular bone defects. The soft tissues were repositioned and sutured with silk sutures to achieve primary closure. Each rat received an intraperitoneal injection of antibiotics post-surgery.

#### 2.11.3 Oxidative stress level

After 2 days post-surgery, dihydroethium (DHE, 25 mg/kg Sigma, USA) fluorescence dye was intravenously administered into the SD rats to investigate the ROS level around the mandibular bone defects according to previous study [51]. After one day, the rats were sacrificed, and the harvested mandibular bones were removed, followed by decalcification procedure under dark condition. Subsequently, the mandibular bones were dehydrated with gradient sucrose solution and embedded by optimum cutting temperature compound (SAKURA Tissue-Tek®, Japan) in a cryostat (Leica, German). Finally, the samples were sectioned, observed using CLSM (Olympus, Japan). DAPI was used for staining nuclei.

#### 2.11.4 Micro-CT evaluation

After 4 and 8 weeks post-surgery, the rats were sacrificed, and the harvested mandibular bones were scanned using micro-CT (Skyscan 1076, Kontich, Belgium). The scanning was performed at a resolution of 18 μm and the images were acquired to reconstruct tomograms with 3D creator software. The bone volume/tissue volume (BV/TV) ratio and BV were measured using CTAN image software based on the micro-CT images.

#### 2.11.5 Histological analysis

Following micro-CT analysis, the obtained mandibular bones were fixed in a 10% neutral buffered formalin solution. Then, rat mandibular bones were decalcified and paraffin embedded. Histomorphological analysis was performed on 5 μm thick sections of the central part of the mandibular bone defects. The sections were stained with Hematoxylin and Eosin (H&E) staining, and images were taken with a microscope (Leica, Germany). Standard protocols were followed for Mason's Trichrome staining. For immunohistochemistry (IHC), after rehydrated slides were further subjected to antigen retrieval for 30 min at 95 °C. Then slides were subsequently incubated overnight at 4 °C with specific first antibodies, or with IgG as negative control (Fig. S13). At the second day, slides were washed in fresh PBS three times and then were incubated using specific second antibodies from HRP-DAB Kit (R&D System). Hematoxylin was used for counterstaining.

### 2.12 Statistical analysis

The data were analyzed by one-way analysis of variance (ANOVA) followed by Tukey multiple-comparison post-hoc test to determine the significance of difference between the test groups. Quantitative data are expressed as mean ± s.d. and the data were indicated with (*) for probability less than 0.05 (*p* < 0.05), (**) for *p* < 0.01, and (***) for *p* < 0.001, respectively.

## 3 Results and discussion

### 3.1 Fabrication and characterization of PDA, PDAH and BPDAH nanoparticles

**Fig. 1:**
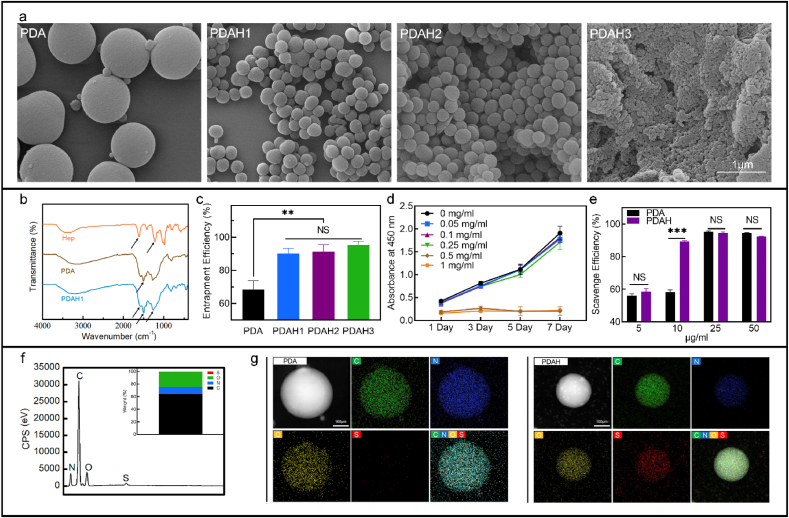
**Characteristics of PDA-based nanoparticles.** (a) SEM image of PDA and PDAH nanoparticles, (b) FTIR spectra of heparin polymer, dopamine, PDA and PDAH nanoparticles, (c) BMP-2 entrapment efficiency of PDA and PDAH nanoparticles, (d) CCK-8 assay results of MC3T3 cocultured with different concentration of PDAH nanoparticles for 1–7 days, (e) DPPH scavenging ability of different concentration of PDA and PDAH nanoparticles, (f) EDS spectra and quantitative analysis of C, N, O and S elements of PDAH nanoparticles, (g) EDS mapping results of PDA and PDAH nanoparticles.

PDA based NPs were synthesized via oxidative self-polymerization of dopamine molecules in water-ethanol mixture. The PDA particles were first investigated by the field emission scanning electron microscopy (SEM). Typical images indicated that the resultant PDA nanoparticles held a monodisperse spherical structure with an average diameter of about 980 nm (Fig. 1a). Upon the addition of heparin (0.5 or 1 mg mL^−1^ based on DI-water) to the PDA solutions, the diameter of obtained PDAH spheres significantly decreases to about 260 nm (Fig. 1a). With the further increase of heparin concentration up to 5.0 mg mL^−1^ (based on DI-water), the mixtures tend to form nanoparticle aggregates with the smallest particle size about 50 nm (Fig. 1a). Clearly, the diameter of PDAH spheres decreases with the increase of heparin concentration. The dopamine can react with heparin through the chemical reaction between carboxyl groups or sulfate groups of heparin and amino groups of PDA polymers. The addition of heparin to the dopamine solutions resulted in the formation of an inclusion complex through a strong hydrogen bond and thus decreases the nanoparticle size.

Results of Fourier transform infrared (FT-IR) spectra provided additional evidence of the successful formation of PDA and PDAH NPs (Fig. 1b). The characteristic peaks observed at 1543-1555 cm^−1^, 1210–1215 and 1625-1630 cm^−1^ were the protonated amino group on PDA, the sulfate ions (-SO_4_^-^) and the carboxylic ions (-COO^-^) on heparin. The ionized PDA can react with heparin through the electrostatic interactions between the negatively charged carboxylic acid salts (-COO^-^) on heparin and the positively charged amino groups (-NH_3_^+^) on PDA according to previous studies [52,53]. The electrostatic interaction between heparin and PDA combined with the polymerization of dopamine induced the self-assembly of PDAH nanoparticles. Furthermore, Energy dispersive X-ray spectroscopy (EDS) equipped in SEM was employed to determine the location of the heparin in the PDAH particles via detection of sulfur element on the sulfated heparin, and the analysis results demonstrating that PDAH nanoparticles contain 73% C, 20% O, 4% N, and 1.2% S (Fig. 1f). The EDS mapping results illustrate that the element of S of the heparin were homogenously distributed inside the PDHA nanoparticles. The uniform distribution of heparin could be beneficial to tether BMP-2 and amplify their activity (Fig. 1g). As heparin concentration increased, the product yield values of PDAH nanoparticles were gradually decreased (Fig. S1b). The yields of the prepared PDA, PDAH1, PDAH2 and PDAH3 were 67.4, 57.7, 35.4 and 2%, respectively. PDAH1 was selected as the further experiment group because it gave higher product yields and smaller particle size distribution compared to other PDA based particles (Figs. S1a and b). The above characteristic results based on the SEM, FITR, and EDS analyses further evidence the successfully synthesis of the PDAH nanoparticles.

In health conditions, free radicals keep a balance level between generation and elimination. During severe trauma and inflammatory, excessive free radicals including superoxide radicals (O_2_•^-^), H_2_O_2_, hydroxyl radicals (OH•) accumulated in local microenvironments tend to cause oxidative stress and oxidative damage to the cells. PDA nanoparticles are known to possess antioxidant ability in the biological system since their distinct hydroquinone structures, which could be able to prevent oxidative stress and enhance the survival, proliferation and osteoinduction of osteoblasts in the treatment of large bone defects. Here, the ROS scavenging activity of PDA and PDAH particles were determined by free 2, 2-diphenyl-1-picrylhydrazyl (DPPH) radicals scavenging assay. Both PDA and PDA particles show strong antioxidant ability in dose-dependent manner and their DPPH scavenging proportions reached a stable plateau of 90% above 25 μg mL^−1^ (Fig. 1e). Note that the PDAH spheres show significantly higher ROS depletion capacity compared to that of PDA particles at low concentration (10 μg mL^−1^), which might be ascribed to the smaller particle size distribution and more hydroquinone motifs of PDAH particles. On the other hand, our results demonstrate that the PDAH nanoparticles show robust BMP-2 binding *in vitro*. In details, the BMP-2 absorption efficiencies on the PDA and PDAH nanoparticles were 68.2% and 90.03%, respectively (Fig. 1c). And per gram of PDAH contains 900.3 μg of BMP-2. Heparin functionalized PDA particles can increase growth factor loading capacity, which is consistent with previous studies that heparin-conjugated biomaterials have been confirmed as superior carrier for BMP-2 tethering and sustain release due to the high affinity interactions between growth factors and heparin [[43], [44], [45]]. Taken together, the developed PDAH spheres with excellent antioxidant properties and high BMP-2 affinity could serve as cellular protection agents against oxidative stress for facilitating bone formations.

### 3.2 Mechanical properties of molecular design of hydrogels

**Fig. 2:**
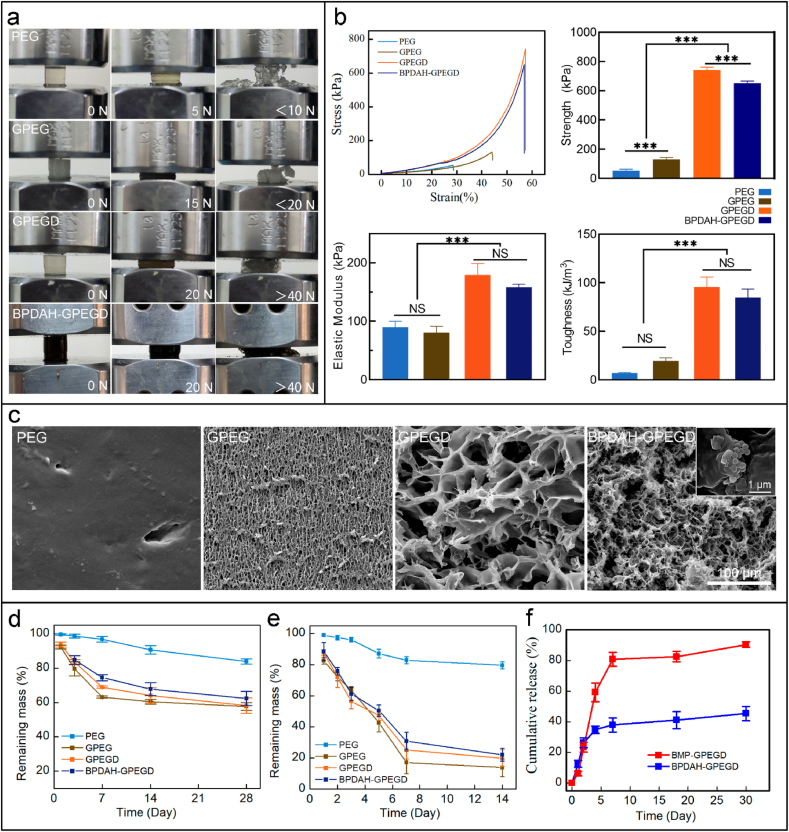
**Characteristics of hydrogels.** (a) Optical images of the various hydrogels under a compression experiment. (b) Quantitative mechanical properties of the molecular designed hydrogels measured by standard mechanical compression tests. (c) SEM images of the cross section of various freeze-dried hydrogels. Degradation properties of different hydrogels in PBS (d) without and (e) within collagenase (12.5 U mL^−1^). (f) Cumulative release of BMP-2 from BPDAH-GPEGD and BMP-GPEGD hydrogels.

The addition of trace amounts of 2-(dimethylamino)ethyl methacrylate (DMAEMA) in the hydrogels showed a significant increase in mechanical properties. During compressive test of various hydrogels, the PEG and GPEG gels were fractured at low compressive forces <20 N, whereas the molecular design of GPEGD and BPDAH-GPEGD hydrogels still retained the integrative structures at high compressive forces of >40 N (Fig. 2a). To quantify the addition of DMAEMA on the mechanical properties, various hydrogels samples were subjected to uniaxial compressive testing. Six concentrations of DMAEMA were introduced to the GPEG hydrogels, and the mechanical results indicate that the addition of 0.5% of MDAEMA to the GPEG hydrogels shows the highest compressive strength, elastic modulus and toughness than those of other groups (Fig. S4). Thus, the 0.5% of MDAEMA were used for functionalization of GPEG hydrogels in our further study.

The representative compressive stress-strain curves are shown in Fig. 2b, and the ultimate compressive strength, elastic modulus and toughness are summarized in Fig. 2b and Table S2. The compressive strength, elastic modulus, and toughness for the GPEGD hydrogels were remarkably higher than those of the PEG and GPEG hydrogels. Quantitatively, the compressive strength of GPEGD and BPDAH-GPEGD hydrogels was significantly improved to 742 ± 19.22 kPa and 651 ± 15.08 kPa compared to 131.33 ± 10.87 kPa of the GPEG hydrogel. Besides, the elastic modulus of the GPEGD and the BPDAH-GPEGD hydrogels sharply increased to 179.24 ± 19.22 kPa and 158.33 ± 5.08 kPa, respectively, which were ∼2.5 times higher than that of the GPEG hydrogel (80.42 ± 10.88 kPa). Consequently, compressive toughness of the GPEGD and the PDAH-GPEGD hydrogels were markedly increased to 95.73 ± 10.15 kJ/m^3^ and 84.98 ± 8.62 kJ/m^3^ compared to 19.69 ± 2.94 kJ/m^3^ of the GPEG, accounting for an increase of ∼5 times.

**Scheme 1:**
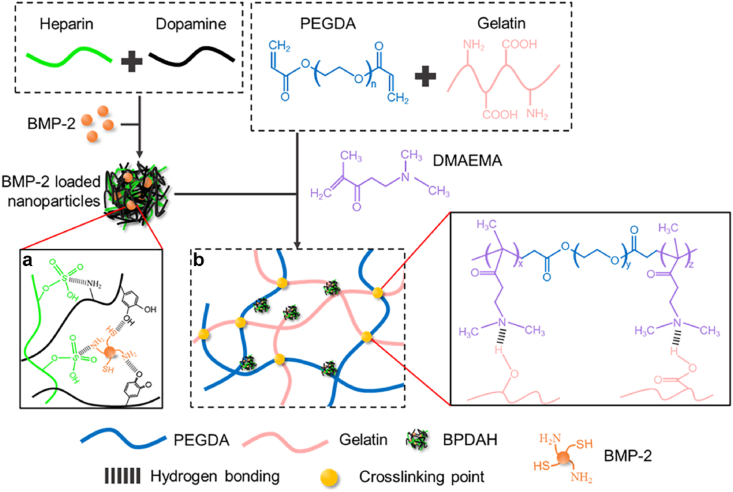
**Multiscale design of stiffening and ROS scavenging hydrogels for bone regeneration.** (a) Preparation of BPDAH nanoparticles via an oxidation and self-polymerization method. (b) Molecular design of stiffening hydrogels for incorporation of the bioactive BPDAH nanoparticles for the augmentation of bone tissue regeneration.

A trace amounts of the DMAEMA additive to the GPEG hydrogels shows the highest compressive stress, modulus and toughness. This is mainly due to the fact that the DMAEMA molecules can physically and chemically interacted with gelatin and PEGDA polymers in the molecular design of hydrogels inspired by our previous study [54] (Scheme 1b). In details, once the DMAEMA monomer was introduced into the gelatin and PEGDA mixtures, the –N(CH_3_)_2_ groups in DMAEMA molecules immediately target to the carboxyl groups or hydroxyl groups of the gelatin polymers and form strong hydrogen bonds. In addition, the methacrylate groups on the other end of DMAEMA can covalently polymerized with PEGDA through free radical polymerizations. Notably, as increasing the DMAEMA concentration from 0.5 wt % to 1 wt % in the GPEGD hydrogels, the ultimate compressive stress, elastic modulus and toughness markedly reduced to 188.89 ± 14.65 kPa, 91.76 ± 14.38 kPa, 18.07 ± 7.56 kJ/m^3^, which was similar to that of the GPEG networks (Table S2). It is worthy to notice that the increasing amounts of DMAEMA might inhibit the degree of polymerization of PEGDA, resulting in poor mechanical behaviors due to the weak covalent structures, which was consistent with our previous results [54]. Therefore, the trace amounts of poly DMAEMA (PDMAEMA) in these systems, serving as bridges with one end covalently cross-linked to PEGDA chains while the other end physically interact with the carboxyl or hydroxyl groups of gelatins, can synergistically improve the mechanical properties of the molecular designed hydrogels by the balance of hydrogen bonds and covalent structures in the GPEGD networks.

### 3.3 Degradation and swelling properties of various hydrogels

As shown in Fig. 2c and Fig. S3b, the molecular design of GPEGD and PDAH-GPEGD hydrogels exhibited an interconnected porous structure (∼10.7 μm pore size), which is highly than that of the PEG (dense surface topography) and GPEG hydrogels (∼3.8 μm pore size). The pores of hybrid hydrogels became larger and looser with the addition of gelatin and DMAEMA molecules. The highly porosity of the GPEGD hydrogels could be ascribed to the lower polymerization of PEGDA in the PEGDA/gelatin networks upon the addition of trace amounts of DMAEMA molecules. Specifically, a large number of BPDAH nanoparticles were observed on the edge of pore surfaces in the BPDAH-GPEGD hydrogels.

The physical properties of composite hydrogels, particularly biodegradation and swelling properties are important in evaluating their potential for promoting bone regeneration. The weight loss ratio of various hydrogels in PBS at 37 °C was investigated, revealing that the hydrogels undergo degradation over time (Fig. 2d). The pristine PEG hydrogels were relatively stable in PBS solutions, and more than 83% of them were remained after 28-day incubation. Furthermore, after introduction of gelatin in the PEG hydrogels, the degradation rates of GPEG hydrogels were markedly increased, and about 40% of them were degrade after 28-day incubation in PBS. When the DMAEMA molecules and BPDAH nanoparticles were incorporated in the GPEG hydrogels, there is no significantly change of degradation of GPEGD and BPDAH-GPEGD hydrogels compared to that of GEPG hydrogels. Note that the GPEG based hybrid hydrogels present fast degradation rates in the presence of collagenase (Fig. 2e), revealing that less than 30% of them were remained after 14 days. Besides, the swelling ratio significantly declined to about 590% along with the addition of gelatin compared to that of pure PEG hydrogels (∼840%) (Fig. S3c), suggesting that the GPEG, GPEGD and BPDAH-GPEGD hydrogels exhibited a better anti-swelling properties than that of the PEG hydrogels. A possible explanation for the observed phenomenon is that the addition of gelatin may enhance the hydrogen bonds in the hybrid networks.

The degree of degradation of polymer hydrogels mainly depends on chemical structures (molecular weight, functional groups) and the crosslink types of the polymer networks. In addition, the elastic modulus of hydrogels relies on the crosslink types (covalent bonds, hydrogen bonds, host-gust chemistry, ionic interactions, hydrophobic interactions and others) and densities of the crosslinks. Here, the elastic modulus of PEG hydrogels mainly derived from the covalent crosslinks of -C-C- bonds between PEG chains, and the elastic modulus of GPEG hydrogels are attributed to the less covalent crosslinks of -C-C- bonds and a large amounts of hydrogen bonds between gelatin polymers. The hydrolysis of covalent -C-C- bonds cannot take place in physiological conditions, but the hydrogen bonds between gelatins can be cleaved by water in physiological environments. Besides, the peptide bonds in the gelatin polymers increases their enzymatic hydrolysis of GPEG. Thus, although the elastic modulus of PEG and GPEG is similar in our work, but the degree of degradation of GPEG is higher than that of the PEG hydrogels. Furthermore, the addition of DMAEMA into the GPEG hydrogels significantly enhances their hydrogen-bond densities between PEG and gelatin polymers through DMAEMA build blocks. Meanwhile, the sum of the covalent -C-C- bonds in the GPEG and DAMEMA hydrogels are similar since only trace amounts of DMAEMA additives were introduced to the GPEG hydrogels. Thus, the degradation kinetics of both hydrogels mainly depends on the concentrations of gelatins in each network.

Specifically, achieving anti-swelling hydrogels need subtle design strategy via precise regulation of the degree and types of crosslinking bonds based on hydrophilic groups in the hydrogels. In general, the enhancement of hydrogen bonds or ionic coordination interactions between hydrophilic groups (carboxyl groups, hydroxyl groups, amino groups and so on) and other additives (functional polymers, nanoparticles or cationic ions) play a vital role in inhibiting water-hydrophilic interactions which achieves antiswelling properties for hydrogels. For example, Li et al. [55] have constructed antiswelling poly (acrylamide-co-AA)/sodium alginate hydrogels by forming multiple crosslinked types such as polymer entanglements, strong hydrogen bonds,and carboxyl groups-Fe^3+^ cooperation bonds. Qiu et al. [56] reported a rational design of antiswelling and strong polyvinyl alcohol (PVA) exogels by solvent-exchange strategy, where the outstanding antiswelling property of the gels was ascribed to the high crosslinking densities stemming from homogenized polymer networks and strong hydrogen bonds of hydroxyl groups. Besides, recent study have demonstrated that the coordination bonds between high valence Al^3+^ of organic nanoparticles and carboxylic groups of polymer endows hydrogels with excellent antiswelling ability [57]. Thus, we proposed that the enhancement of antiswelling mechanism of our GPEGD is ascribed to the enhancement of hydrogen bonds interactions between gelatin and DMAEMA molecules in the hybrid networks.

The BMP-2 release kinetics of tissue engineering scaffolds play a pivotal role in modulating bone regeneration. As shown in Fig. 2f, direct BMP-2 encapsulation in the GPEGD hydrogels showed initial burst release in which more than 80% of total BMP-2 were delivered in the first 6 days. On the contrary, BPDAH-GPEGD presented sustain release of BMP-2 for a long-term period where less than 50% of total BMP-2 were released after 30 days, which demonstrated that PDAH nanoparticles could serve as a favored carriers for BMP-2 loading and release.

### 3.4 *In vitro* ROS scavenging ability of hydrogels

**Fig. 3:**
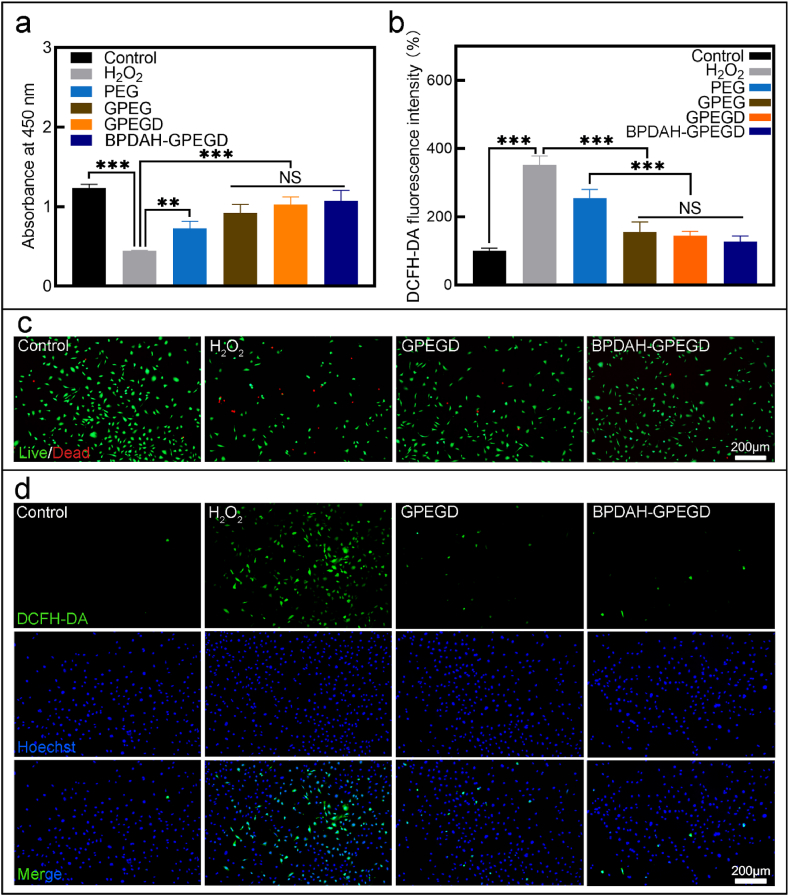
**Antioxidant activity of hydrogels.** (a) CCK-8 assay results and (b) ROS fluorescence intensity of MC3T3 cocultured with different hydrogels under H_2_O_2_ conditions. (c) Representative Live/Dead staining images of MC3TC after coculture with different hydrogels for 10 h indicated that the rescue effect of GPEGD and BPDAH-GPEGD hydrogels for MC3T3 cell under H_2_O_2_ environment. (d) Epifluorescence images of DCFH fluorescence intensity of MC3T3 treated with different hydrogels demonstrated that the GPEGD and BPDAH-BGPEGD hydrogels could significantly deplete the intracellular ROS content of MC3T3 under oxidant stress conditions. Green fluorescence indicates DCFH, which is the product resulting from the reaction between ROS and DCFH-DA indicator. Nuclei were stained with Hoechst 33342 (blue).

We further examined the protective effect of hydrogels in MC3TC cells under ROS damage environments. When the cells were co-cultured with H_2_O_2_, a large number of cells were dead, implying that the intracellular ROS content significantly decreased the cell viability of MC3T3. When the H_2_O_2_ treated cells were supplemented with hydrogels, the cell viability was recovered (Fig. 3a, c and Fig. S4). Noticeably, the highest cell viability (86.9% compared to the control group) was observed on the BPDAH-GPEGD hydrogels under ROS conditions, suggesting that the addition of BPDAH significantly increased the antioxidant ability of the molecule designed hydrogels. The intracellular ROS was visualized using dichlorofluorescein diacetate (DCFH-DA) as an ROS-sensitive cell-permeable fluorogenic marker. To validate the ROS scavenging of hydrogels, MC3T3 cells were incubated with H_2_O_2_ for 1 h with the treatment of different types of hydrogels. When treated with H_2_O_2_, the ROS intensity of MC3T3 increased compared with the control group (normal growth media), indicating the elevated intracellular ROS (Fig. 3b, d and Fig. S5). After the addition of hydrogels to the H_2_O_2_ treated cells, the DCFH-DA intensity of MC3T3 decreased significantly compared with H_2_O_2_ group, indicating the *in vitro* protection effect of hydrogels against ROS. Most importantly, the lowest intensity was achieved on the BPDAH-GPEGD hydrogels compared to other hydrogels.

As mentioned above, the PDAH nanoparticles could further enhanced the ROS scavenging ability when the cells were treated with increasing ROS levels. As shown in Fig. 3, the GPEGD gels could rescue the cell viability and deplete most of the ROS against 400 μM H_2_O_2_ treatment. However, as increasing the concentration of the H_2_O_2_ to 800 μM, the ROS capacity of GPEGD was not enough to rescue the cell viability or normal functions (Fig. S7). Thus, the PDAH nanoparticles not only plays an important role in mediating BMP-2 release, but also serves as robust antioxidant agent to deplete excessive ROS levels *in vivo* for synergistically promoted bone formations.

### 3.5 In vitro osteoinduction of various hydrogels

**Fig. 4:**
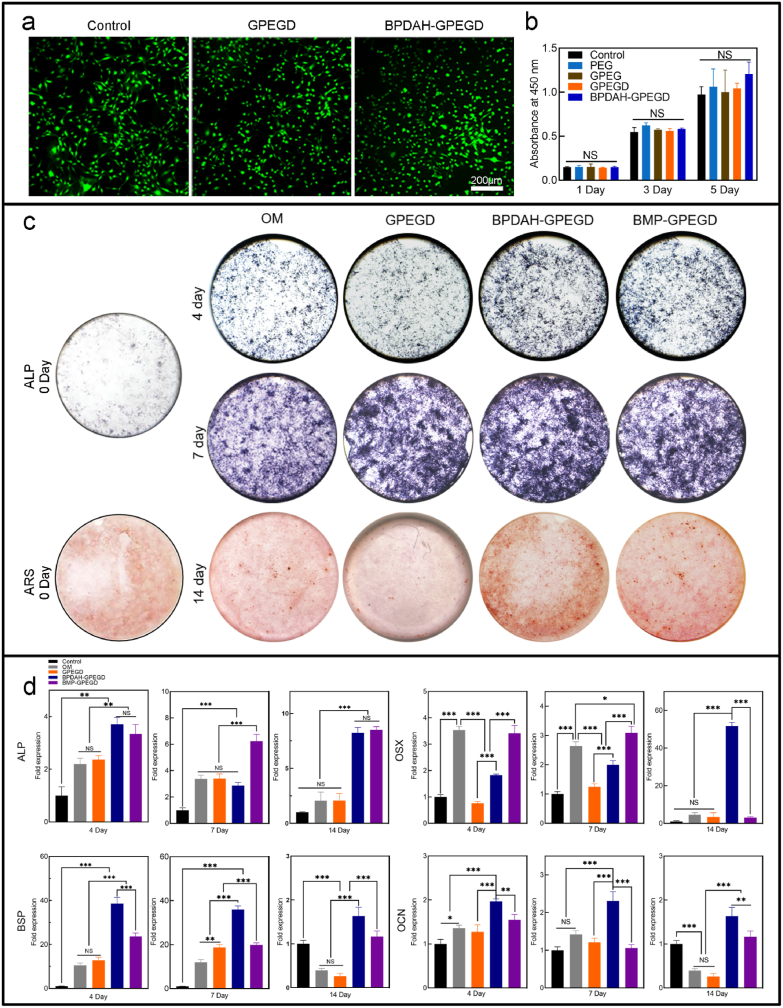
***In vitro* cell adhesion, proliferation, and differentiation on hydrogel and multiscale design of stiffening and bioactive hydrogels.** (a, b) All hydrogels supported initial MC3T3 adhesion/spreading (day 1) and proliferation, as determined by Live/Dead imaging and CCK8 assay of cells seeded on hydrogel surfaces. TCPS acted as the positive control. (c) The ALP and ARS staining of MC3T3 after cocultured with different hydrogels on days 4, 7 and 14. (d, e) Osteogenic gene expression (ALP, BSP, OSX and OCN) of MC3T3 in control, GPEGD, BPDAH-GPEGD and BMP-GPEGD group on days 4, 7 and 14 (day 0 as control).

The ability to support initial cell adhesion and subsequent proliferation is an important requirement of a tissue-engineered scaffold. MC3T3 preosteoblasts were used to investigate the effect of hydrogels on initial cell adhesion and proliferation. Compared to single network PEG gels, gelatin-based gels preferred to facilitate cell adhesion since gelatin is a denatured protein that contains RGD groups for mediating cell adhesion performance via integrins [4]. The cells readily adhered to GPEG hydrogels, and the addition of DMAEMA molecules and BPDAH nanoparticles to the GPEG gels did not result in any significant influence in initial cell adhesion (Fig. 4a). The cellular morphology indicated that the GPEGD and nanocomposite BPDAH-GPEGD gels are cytocompatible and did not elicit any cytotoxic effects.

Herein, the MC3T3 cells spreading well on both biopolymer gelatin incorporated GPEGD and GPEGD gels, and the composition effects might be good enough to block the stiffness effects on the cell spreading and adhesion. Cell spreading behaviors on the biomaterial substrates depend on both physical and chemical cues. The optimum stiffness for different cell lines to form their functions is varied. Recently study even indicated that even the soft substrates within biopolymers can facilitate cell spreading than that of the stiffer substrates without biopolymer modification [58]. Beside stiffness, cells also respond to structural cues such as mesh size, chemical composites, and degradation properties. Thus, the higher modulus of GPEGD hydrogels did not promote cell spreading and adhesion might be ascribed to the adhesive biopolymers and suitable modulus of the GPEG gels. The CCK-8 results indicated that all gels supported cellular proliferation, and cells showed metabolic activity over a period of 5 days similar to that of the control group (Fig. 4b). These results indicated that stiffening GPEGD and nanocomposite BPDAH-GPEGD gels are cytocompatible.

Specific physical combined biochemical cues of bone implants indeed play a critical role in regulating cellular processes and their fate. Specifically, the optimization of strengthened mechanical stimuli, antioxidant properties and sustain release of growth factor within tissue engineered scaffolds might enhance tissue regenerations [[59], [60], [61]]. To further understand whether the stiffening and ROS-scavenging gels functionalized with biomolecules in different adsorption manners have a pivotal role on mediating cell differentiation, we next evaluate the osteoinduction abilities of GPEGD, BPDAH-GPEGD and BMP direct loaded to GPEGD gels (BMP-GPEGD) on MC3T3 cells *in vitro*. The effects of BMP-2 and BPDAH encapsulated in GPEGD on osteogenic responses were conducted by qualitative and quantitative assessment in different periods. First, ALP and ARS staining was used to detect the degree of osteogenic differentiation in MC3T3 seeded on various hydrogels *in vitro* (Fig. 4c). The results indicated the addition of BMP-2 and BPDAH was able to significantly enhance the ALP activity of the GPEGD hydrogels. More importantly, ARS results showed much darker red stains was observed in the BPDAH-GPEGD hydrogels, indicating their remarkable osteoinduction capability for producing mineralized matrix. Next, qPCR analysis of differentiated MC3T3 at day 4, 7 and 14 revealed that the expression of osteogenic differentiation markers ALP, OSX, OCN and BSP was relatively higher in the BMP-2 encapsulated hydrogels than that of pristine GPEGD gels (Fig. 4d). In details, both BPDAH-GPEGD and BMP-GPEGD hydrogels promoted the expression of transcription factors (OSX) as well as osteoblast marker genes (ALP, BSP, OCN) compared with the GPEGD hydrogels after incubation for 14 days (Fig. 4d). Moreover, BPDAH-GPEGD hydrogels showed the higher expressions of OSX, BSP and OCN than the other groups at 14 days, indicating that PDAH carriers incorporated in hydrogels for sustain release of BMP-2 showed facilitating effects for enhanced osteo-differentiation ability. These results demonstrated that the nanocomposite BPDAH-GPEGD gels may be considered biomimetic microenvironments to control cellular behaviors and osteoblast differentiation.

Our *in vitro* results demonstrated that the sustain release of BMP-2 of the stiffening and ROS-scavenging GPEGD hydrogels through the PDAH nanocarriers play a key role in promoting cell osteoblast differentiation. As we known, direct administration of BMP-2 with hydrogels for bone regeneration could cause a series of clinical side effects such as inflammation, osteoclast activation, and bone cyst formation. Thus, extensive efforts have been made to develop of multiple strategies, such as direct covalent grafting [62], biomimetic nanoparticles [63], gelatin microspheres [32], or stimuli-responsive carriers [64], for spatiotemporal control of BMP release in various hydrogels to enhance their bioactivity. In consistent with previous findings, the current results indicated that BMP-2 protected by PDAH nanoparticles also showed a more sustained release profile in the BPDAH-GPEGD gels since the slow degradable PDAH nanoparticles could strongly adhere to various polymer hydrogels through their catechol chemistry [46,47]. More importantly, BPDAH decorated GPEGD hydrogels maintained high bioactivity for initiating cell differentiation and mineralization in a long-term, which might be ascribed to the facts that heparin chains in the PDAH nanoparticles exhibit strong binding affinity to BMP-2.

### 3.6 Mandible bone repair with stiffening and bioactive hydrogels

According to previous studies [65,66], we detect the ROS level of hard tissues treated with various hydrogels by DHE staining. We used mandibular bone defects model to evaluate the ROS scavenging capabilities of hydrogels for hard tissues. As shown in Fig. S9, the control and PEG groups had significantly stronger ROS fluorescence intensity than the other three groups after 3 days post-surgery implantation. Note that the addition of gelatin biopolymer significantly enhances the antioxidant properties of the PEG hydrogels, while the introduction of PDAH nanoparticles to the GPEGD group did not remarkedly enhance their ROS scavenging ability, which might be ascribed to the slow permeating of PDAH nanoparticles to hard bone tissues. Collectively, the antioxidant ability of GPEG or GPEGD hydrogels might be able to maintain the redox balance in a short-term period in bone defects model. However, as bone regeneration is a step-wisely dynamic process with accumulative production of large amounts of ROS levels, the robust ROS antioxidant ability of PDAH-GPEGD hydrogels might be necessary for depletion of excess ROS level around defect sites to enhance new bone formation.

**Fig. 5:**
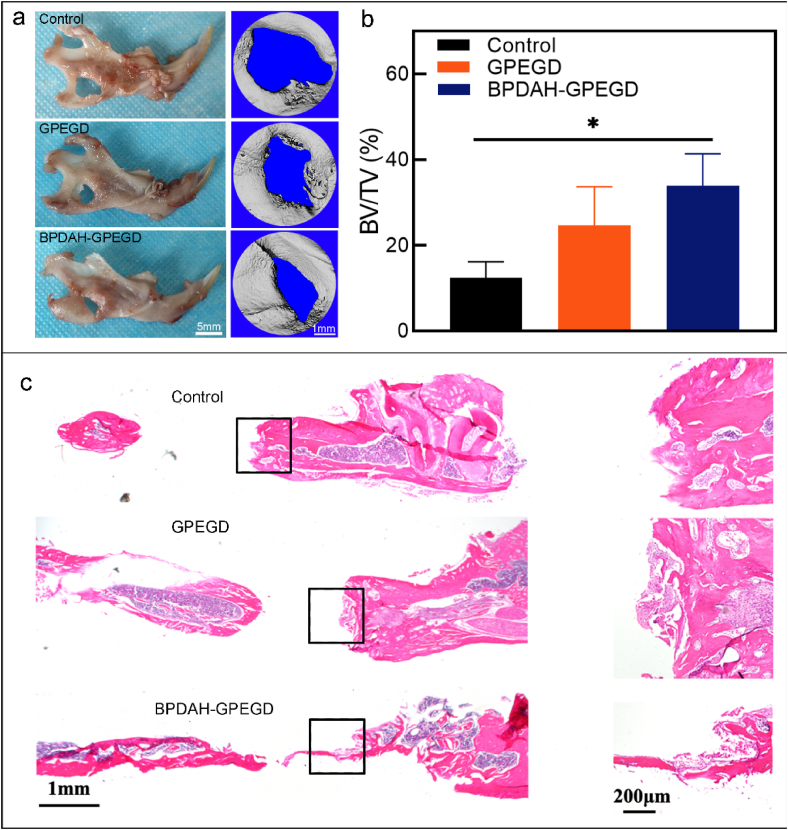
***In vivo* osteogenesis performance of GPEGD and BPEGD-GPEGD hydrogels after 4 week-implantation.** (a) Representative optical images and Micro-CT images, and (b) quantitative analysis of the BV/TV of newly formed bone tissue in harvested mandibles obtained from SD rats after treatments for 4 weeks. (c) H&E-stained histologic sections of mandible decalcified sections.

Next, *in vivo* bone regenerative capacity of the ROS scavenging GPEGD, BPDAH-GPEGD hydrogels were evaluated using a well-established critical mandible defects model in rats. During 4 weeks of the experiment, none of the rat showed any side effects and complications such as inflammatory soft tissue swelling, infection or ectopic bone formation (Fig. S10 and Fig. S11). As shown in Fig. 5, newly generated bone in the original defect area was observed for the GPEGD groups, and was significantly enhanced with BPDAH nanoparticles, whereas no obvious regenerated bone was found in the control group. When the remaining defect area was normalized with the original defect area in Fig. S7b, the bone healing area of BPDAH-GPEGD hydrogels (33.9 ± 7.4%) was considerably increased in comparison with that of the GPEGD hydrogels (24.7 ± 8.9%) and the control group (12.4 ± 3.7%). Furthermore, hematoxylin and eosin (H&E) analysis indicated small amounts of bone tissue and less bone formation in the control groups (Fig. 5c). Bone regeneration efficacy of the BPDAH-GPEDG and GPEGD groups were superior to those of the control group. Moreover, the formation of new osseous tissue was clearly observed for the BPDAH nanoparticles containing GPEGD hydrogels, which is in consistence with the Micro-CT analyses.

**Fig. 6:**
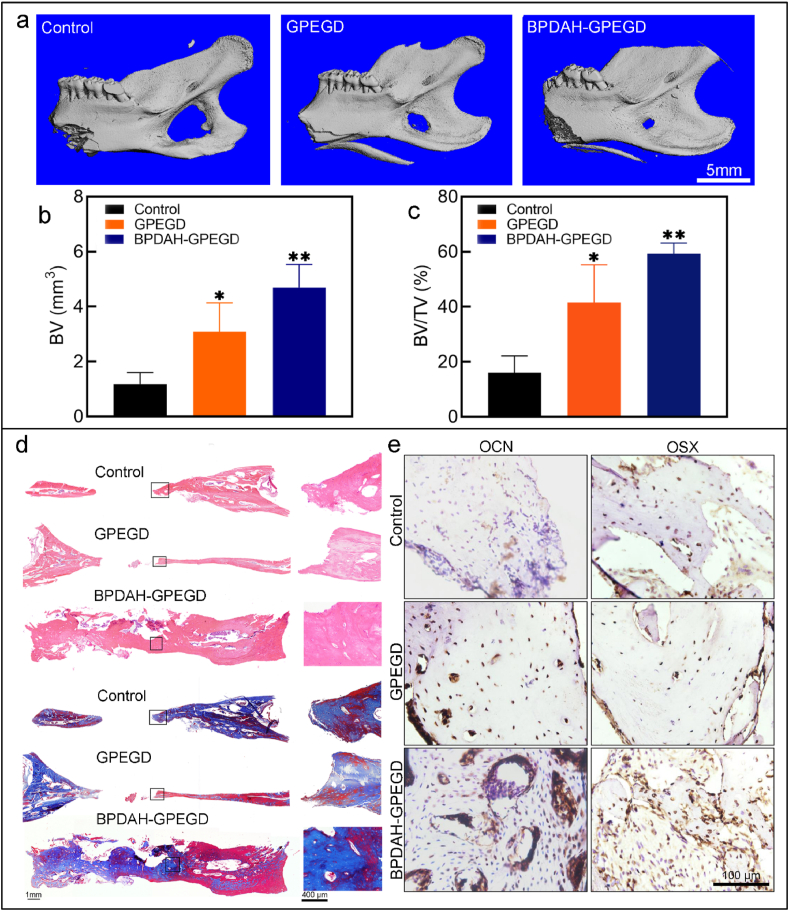
***In vivo* osteogenesis performance of GPEGD and BPEGD-GPEGD hydrogels after 8 week-implantation.** (a) Representative Micro-CT images, and (b) quantitative analysis of the BV and the BV/TV of newly formed bone tissue in harvested mandibles obtained from Sprague-Dawley rats after treatments for 8 weeks. (c) H&E-stained and (d) Masson's trichrome-stained histologic sections, and (e) representative immunohistochemical staining images of OCN and OSX of mandible decalcified sections after 8 weeks of implantation.

After 8-week implantation, Micro-CT and histological analysis shows that all the groups increased the amount of mature bone tissue (Fig. 6). As presented in Fig. 6a, the control group and PEG hydrogels have only a small amount of new bone tissue that is distributed in the central part of the defect regions. More importantly, the bone healing area of mechanical reinforced GPEGD hydrogels (41.47 ± 11.29%) was considerably increased in comparison with that of the GPEG hydrogels (30.34 ± 1.64%). These results were consistent with previous studies. For example, Janorkar et al. [67] documented that cellular mineralization was significantly higher within stiff elastin-like polypeptide/collage hydrogels (E = 35–45 kPa) than all other soft collage hydrogels (E = 4.5–25 kPa). Whitehead et al. [68] also confirmed that stiff PEG hydrogels with higher elastic modulus of 50–60 kPa increased osteo-differentiation compared to soft PEG hydrogels with lower elastic modulus of 8–10 kPa. Xu et al. [69] found that larger volumes of newly formed bone tissue was observed in the stiff graphene/collage aerogels (E = 340–510 kPa), with displaying1.5-fold higher BV and BV/TV ratio than that of other soft groups (E = 200–270 kPa). Taken together, the results demonstrated that the stiffening GPEGD gels with higher modulus promote new bone tissue formation, which give a prospective to engineer biomaterial with large-scale mechanical properties for integration of mineralized bone.

Furthermore, as the ROS scavenging capability of the hydrogels increased, the newly formed bone tissues gradually increased, and the robust ROS scavenging PDAH-GPEGD hydrogels showed highest bone regeneration capability in comparison with the other hydrogel groups without BMP functionalization (Fig. S12). These results suggest that the robust ROS scavenging hydrogels create favorable redox conditions to accelerate in situ bone regeneration. In consistent with previous studies that the introduction of antioxidant components to the bone implants can significantly enhance bone regeneration in a bone defect model [70,71]. The strong antioxidant hydrogels can promptly deplete elevated levels of ROS near mandible defects to reduce the harmful effects such as oxidative stress and cell damage and therefore facilitate bone tissue formation. It was found that there was no significant difference in the newly formed BV and BV/TV between PDAH-GPEGD and GPEGD, suggesting that the antioxidant ability of GPEGD hydrogels might be able to maintain the redox balance in this period. As bone regeneration is a step-wisely dynamic process with accumulative production of large amounts of ROS levels, the robust ROS antioxidant ability of PDAH-GPEGD hydrogels might be necessary for depletion of excess ROS level around defect sites to enhance new bone formation.

Note that the bone healing area of BMP-GPEGD hydrogels (45.16 ± 11.17%) was considerably lower in comparison with that of the BPDAH-GPEGD hydrogels (59.29 ± 3.15%). The bone healing area of BMP-GPEGD hydrogels (45.16 ± 11.17%) was considerably lower in comparison with that of the BPDAH-GPEGD hydrogels (59.29 ± 3.15%) (Fig. S12). Moreover, the BPDAH-GPEGD hydrogels also considerably increased the protein levels of OSX and OCN *in vivo* compared with control and GPEGD groups (Fig. 6e). Although the newly bone tissues in both BPDAH-GPEGD and BMP-GPEGD hydrogels shows no significant differences, the BPDAH nanoparticles endow the GPEGD with better stability of bone formation. Note that the BV and BV/TV of the BMP-GPEGD hydrogels were slightly higher than that of the GPEGD hydrogels, suggesting that the direct delivery of BMP-2 through the GPEGD hydrogels have little positive effects on promoting mandibular bone regeneration. In contrast, the BPDAH-GPEGD hydrogels within sustained release of BMP-2 remarkedly enhanced mandibular bone regeneration. Thus, the PDAH nanoparticles not only increase the antioxidant capability, but also enhance the bioactivity of BMP-2 in the GPEGD hydrogels.

The healing of bone fractures is a complex physiological process that is dominated by essential mechanical, biological and chemical signaling cues of bone implants. Interestingly, our *in vivo* results demonstrated that the sustain release of BMP-2 combined with the stiffening and ROS-scavenging GPEGD hydrogels synergistically enhanced bone regenerations. Upon BPDAH-GPEGD hydrogel implantation to the mandibular defects, the ROS-scavenging functions of the gels began to balance the inflammation environments for normally initiating further process of cell recruitment, osteofunctions and bone reconstructions. Subsequently, the stiffness cues and sustain release of BMP-2 might stimulate differentiation of precursor cells near the defects into secretory osteoblasts and enhance matrix production and the mineralizing deposits of osteoblasts through enhancement of expression of osteogenic proteins. Besides, several important factors such as better matching of the biodegradation rate, cell adhesion motifs and porous structures of the hydrogel might also contribute to mandibular bone regeneration.

Thus, the potential mechanism of our study was proposed as follows. First, we synthesized GPEGD hydrogel with enhanced mechanical properties, and the bone healing area of the mechanical reinforced GPEGD hydrogels was considerably increased in comparison with that of the GPEG hydrogels, suggesting that the enhancement of mechanical properties of hydrogels were conducive for promoting osteogenesis. Second, the addition of gelatin biopolymer, PDAH and DMAEMA into the PEG hydrogels significantly increased their ROS-scavenging ability, which shows a protective effect on cell viability and down-regulated intracellular ROS content. It was reported that elevated ROS are positive modulators of osteoclasts differentiation and maturation, and inhibiting the osteoblast activity, therefore leading to bone loss [72]. Our results found that the robust antioxidant GPEGD and PDAH-GPEGD show fast bone regeneration with stable osteogenic quality. Third, the BPDAH-GPEGD hydrogels present the highest BV of ossified tissue within the mandible defect (4.69 mm^3^) compared to GPEGD hydrogels (3.08 mm^3^) and BMP-GPEGD hydrogels (3.90 mm^3^) after 8-weeks implantation, suggesting that the direct delivery of BMP-2 through the GPEGD hydrogels have little positive effects on promoting mandibular bone regeneration. In contrast, the BPDAH-GPEGD hydrogels within sustained release of BMP-2 remarkedly enhanced mandibular bone regeneration compared to GPEGD hydrogels. Collectively, the BPDAH-GPEGD hydrogels promote the construction and regeneration of mandibular bones by reinforced mechanical properties, enhanced ROS-scavenge abilities and sustained release of bioactive molecules, suggesting their potential therapeutic utility for promoting bone growth in future trials.

## 4 Conclusions

In this study, we first developed novel PDAH nanoparticles via self-polymerization chemistry of phenolic compounds and heparin for BMP-2 adsorption. The introduction of heparin polymers can decrease the particle sizes while increase growth factor loading capacity of the PDA particles. Simultaneously, stiffening and ROS-scavenging GPEGD hydrogels were rationally synthesized through molecular-design strategy. The additions of trace amounts of DAMEMA molecules markedly enhanced the compressive strength, modulus, toughness and antioxidant ability of the GPEG hydrogels. Finally, the GPEGD hydrogels were functionalize with bioactive BPDAH nanoparticles for further enhanced their antioxidant capacity and osteofunctions. Indeed, the antioxidant BPDAH-GPEGD is able to deplete elevated ROS levels to protect cells viability against under high ROS environments. Finally, *in vitro* and *in vivo* results demonstrates that the sustain release of BMP-2 combined with the stiffening and ROS-scavenging GPEGD hydrogels can significantly promoted cell differentiation, mineralization deposits and mandibular bone regenerations. Therefore, our study suggests that these biomimetic hydrogels may be used as a translational potential material to promote the construction and regeneration of mandibular bones.

## Credit Author Statement

L. Ye, and Z. Wang designed the experiments. Y. Wu, X. Li, Y. Sun, and X. Tan performed the experiments. L. Ye, Z. Wang, Y. Wu, X. Li, and, C. Wang collaboratively analyzed the data and wrote the paper. All authors reviewed the paper.

## Ethics approval and consent to participate

All the conducted animal experiments were approved by the Animal Use and Care Committee of Sichuan University (WCHSIRB-D-2020-441).

## Declaration of competing interest

The authors declare that they have no known competing financial interests or personal relationships that could have appeared to influence the work reported in this paper.
