# Supplementary material for: Multiscale design of stiffening and ROS scavenging hydrogels for the augmentation of mandibular bone regeneration
Source: Bioact Mater. 2022 May 23;20:111–25. doi: 10.1016/j.bioactmat.2022.05.021 (PMC9133584; doi:10.1016/j.bioactmat.2022.05.021)
Supplement: Multimedia component 1 [file mmc1.docx]

**Supplementary Data for**

**Multiscale Design of Stiffening and ROS Scavenging Hydrogels for the Augmentation of Mandibular Bone Regeneration**

Yanlin Wu^1,2^, Xuan Li^1,2^, Yimin Sun^1^, Xiujun Tan^1^, Chenglin Wang^1^, Zhenming Wang^1*^, Ling Ye^1*^

^1^State Key Laboratory of Oral Diseases, West China Hospital of Stomatology, Sichuan

University, Chengdu 610041, China

*Corresponding author: Dr. Ling Ye, West China School of Stomatology, Sichuan University, No. 14, 3th Section, South Renmin Road, Wuhou District, Chengdu 610041, China. Telephone: 86-028-85503585; E-mail: yeling@scu.edu.cn;

Dr. Zhenming Wang, West China School of Stomatology, Sichuan University, No. 14, 3th Section, South Renmin Road, Wuhou District, Chengdu 610041, China. Telephone: 86-028-85503585; E-mail: zmwang1220@163.com

^2^These two authors contributed equally to this work.


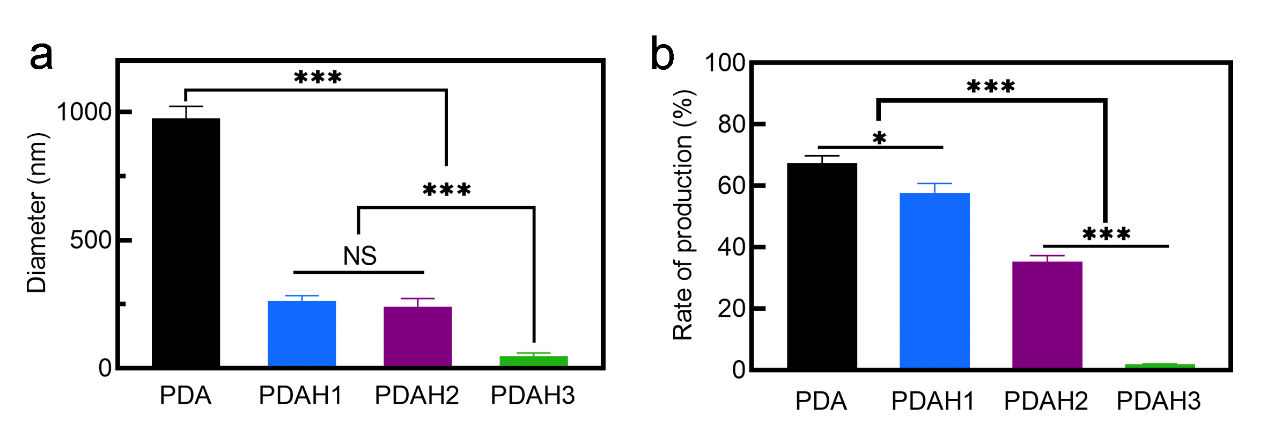


**Figure S1. Characteristic of PDA and PDAH nanoparticles.** (a) Diameter size of PDA and PDAH nanoparticles, (b) Production ratio of PDA and PDAH nanoparticles.


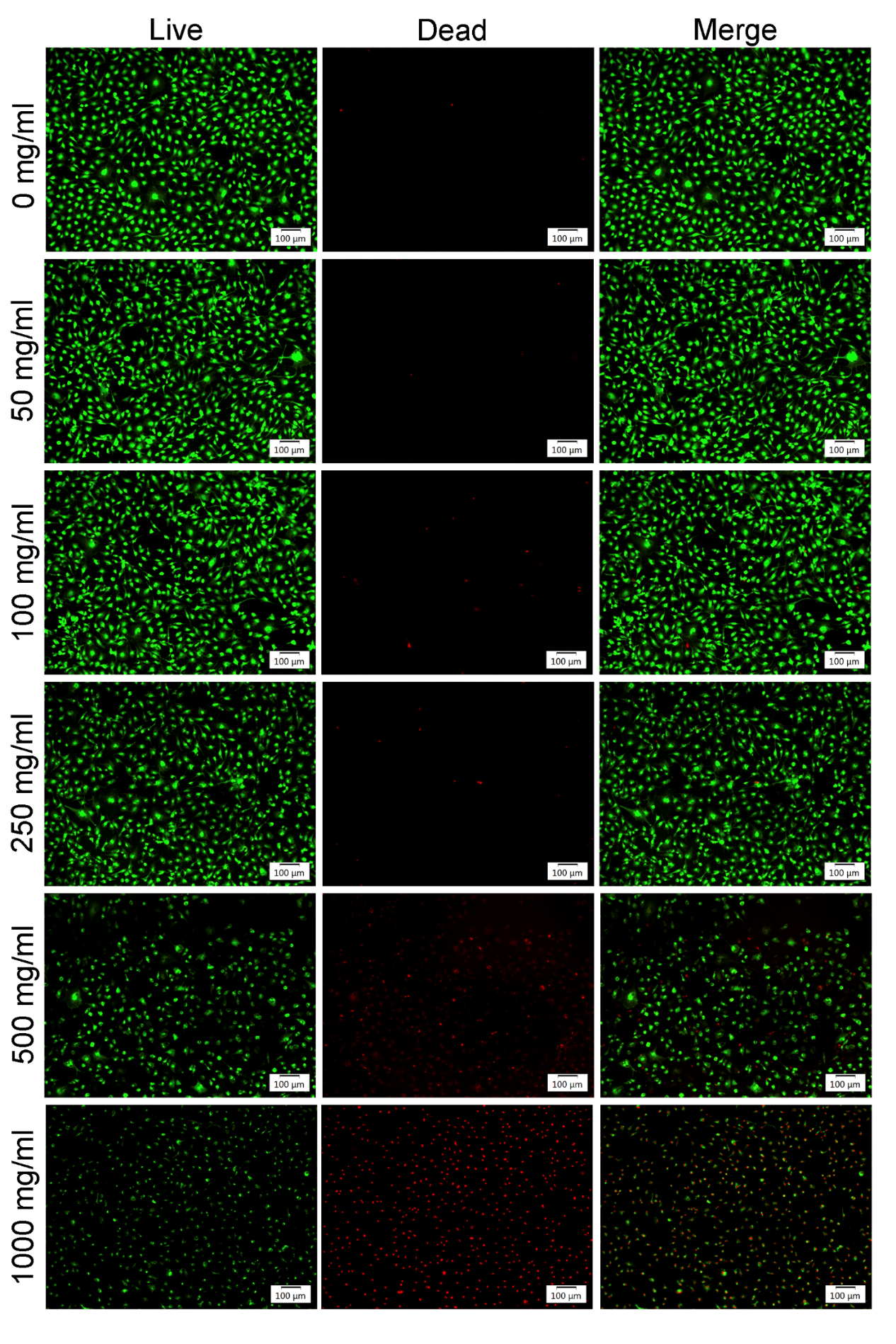


**Figure S2. *In vitro* cell biocompatibility of PDAH nanoparticles.** Representative images of MC3TC after coculture with different concentration of PDAH nanoparticles for 3 days and Live/Dead staining.

**
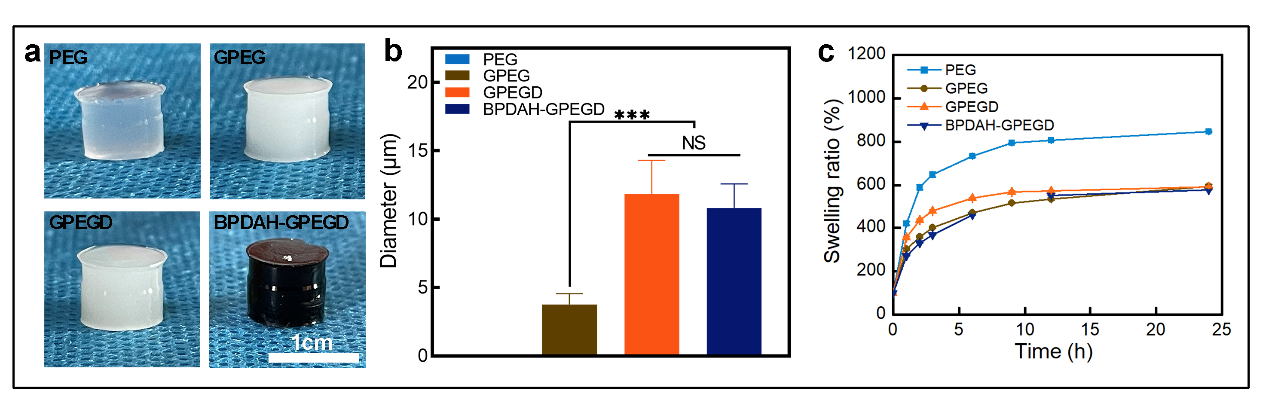
**

**Figure S3. Characteristics of various hydrogels.** (a) Optical images, (b) porous size and (c) swelling properties of PEG, GPEG, GPEGD and BPDAH-GPEGD hydrogels.


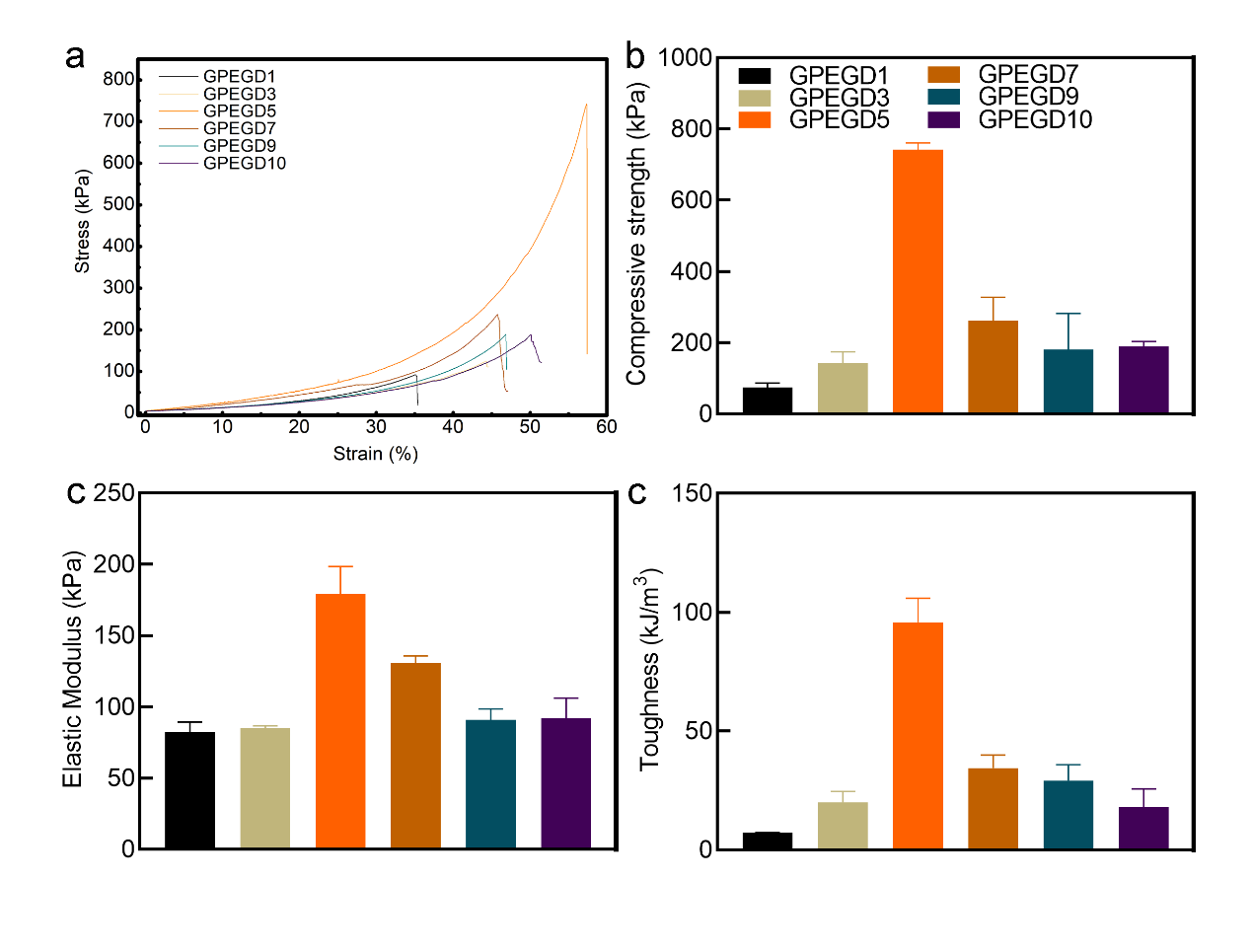


**Figure S4.** **Quantitative mechanical properties of the GPEGD hydrogels with different addition of DMAEMA.** (a) Stress-strain curve, (b) compressive strength, (c) elastic modulus and (d) toughness of PEG hydrogels with different concentrations of DMAEMA.


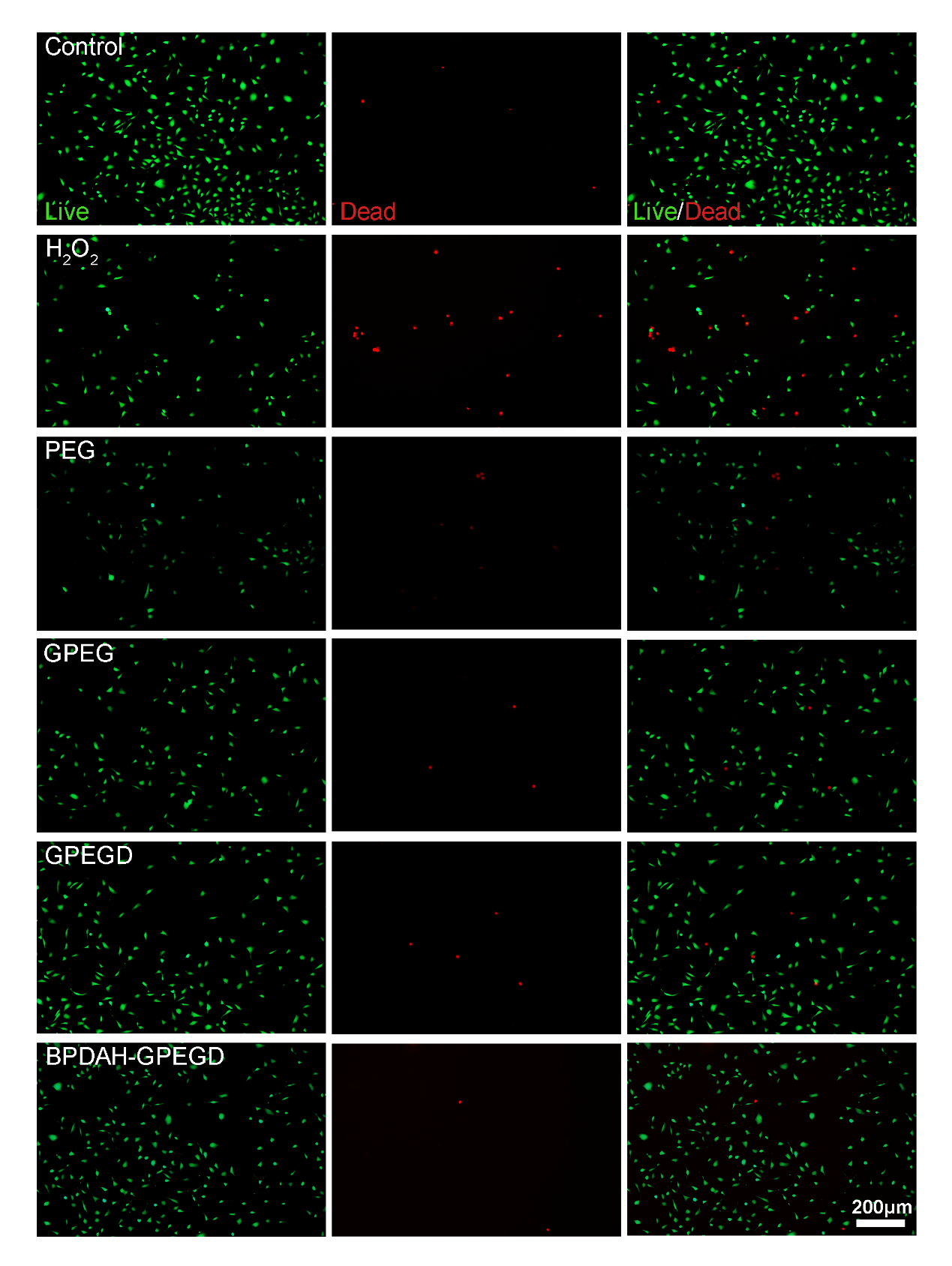


**Figure S5. *In vitro* cell biocompatibility of MC3T3 treated with different hydrogels under oxidative stress environments.** Representative Live/Dead staining images of MC3TC after coculture with different hydrogels for 10 h indicated that the rescue effect of GPEGD and BPDAH-GPEGD hydrogels for MC3T3 cell under H_2_O_2_ environments.


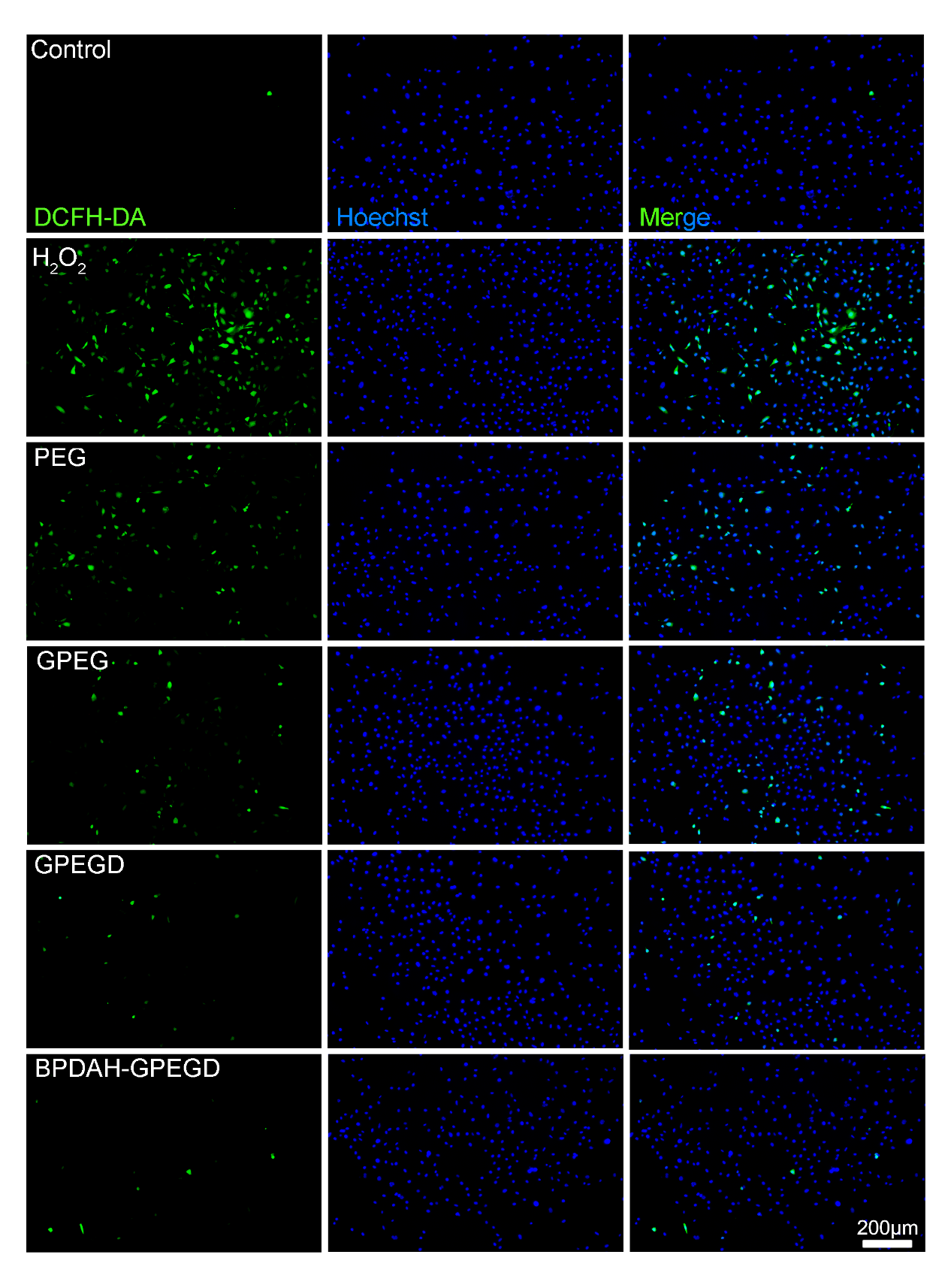


**Figure S6. The rescue effect of different hydrogels for MC3T3 cell under oxidative stress environment.** Representative images of MC3T3 after coculture with different hydrogels for 1 h and DCFH-DA/Hoechst 33342 staining.


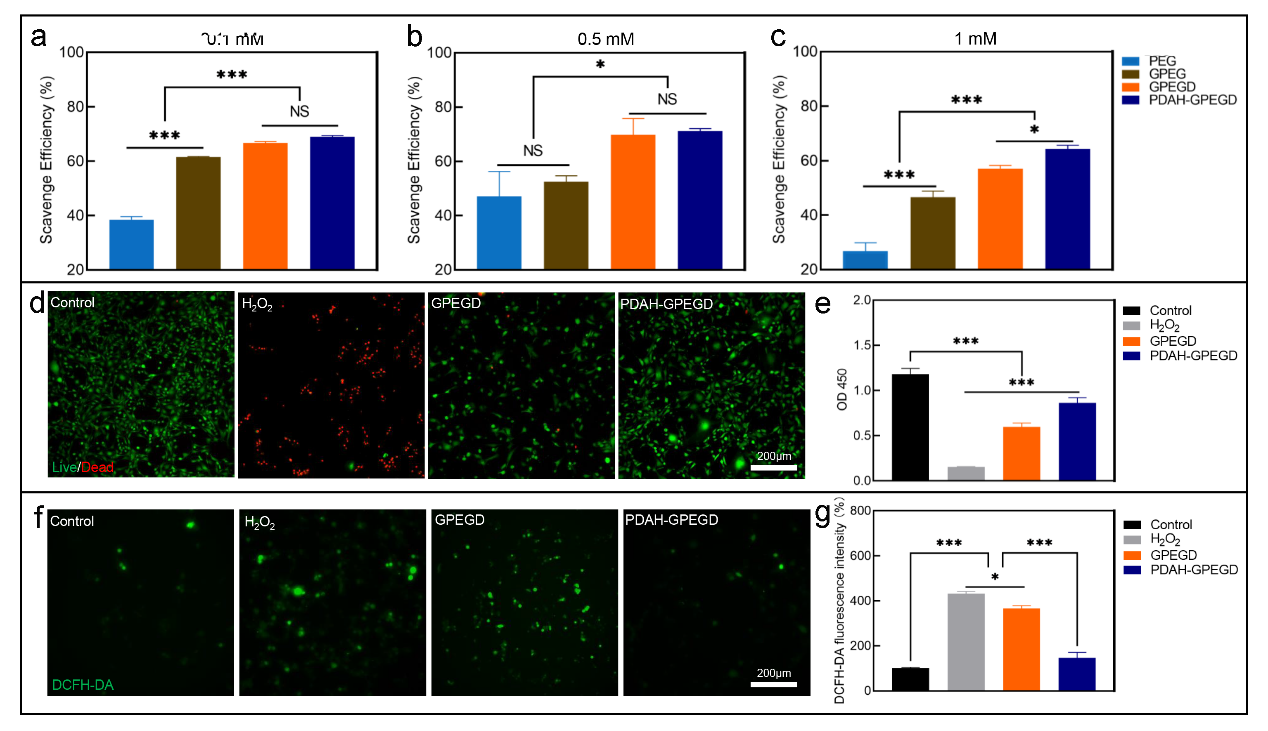


**Figure S7. Antioxidant activity of hydrogels *in vitro*.** Scavenging efficiency of different hydrogels for (a) 0.1 mM, (b) 0.5 mM and (c) 1 mM DPPH. (d) Representative Live/Dead staining images and (e) CCK-8 results of MC3T3 cells after coculture with different hydrogels for 10 h under 800 μM H_2_O_2_ environment. (f) Representative epifluorescence images and (g) DCFH-DA fluorescence intensity of MC3T3 cells treated with different hydrogels for 1 h under 800 μM H_2_O_2_ environment.


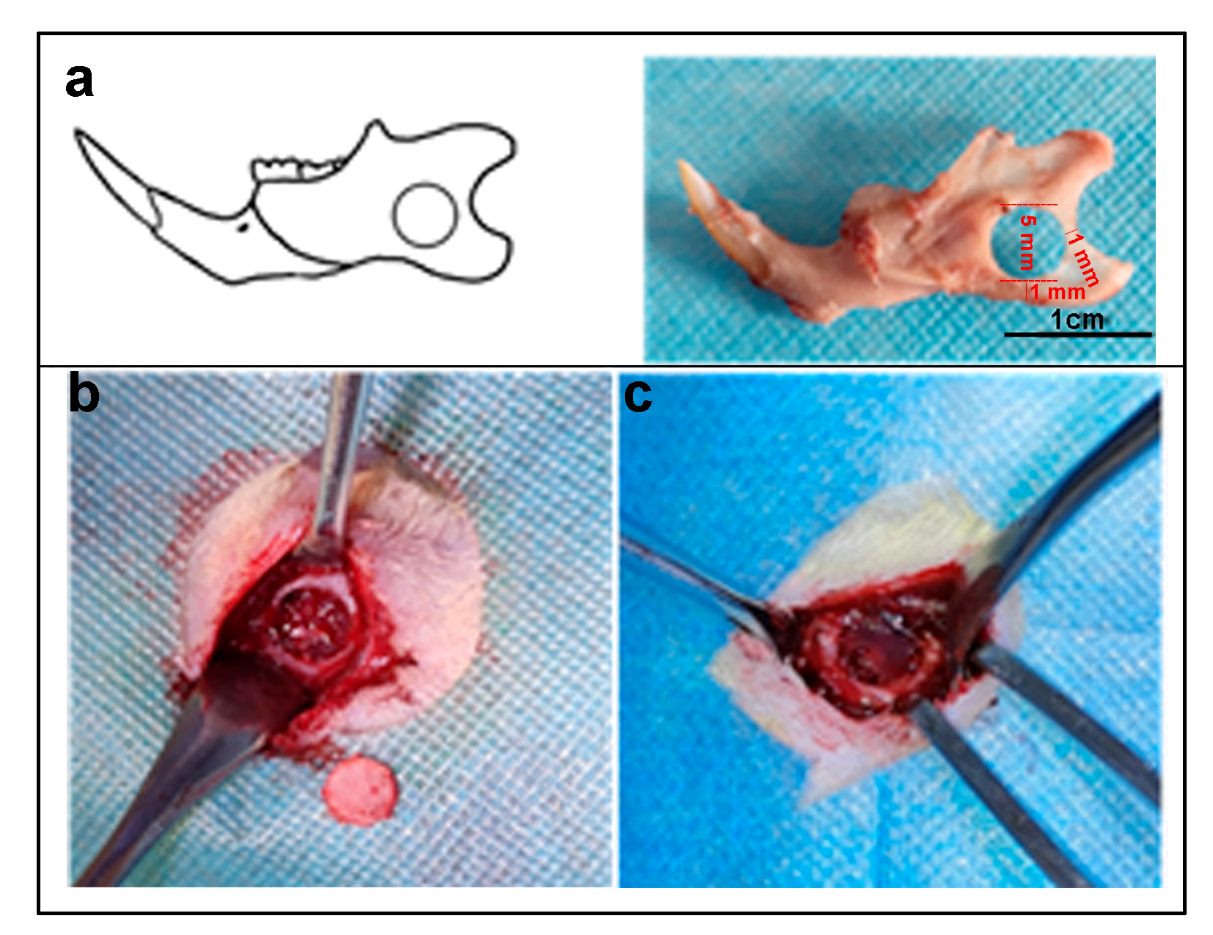


**Figure S8. Typical mandibular bone defect model used in rats for evaluating the osteoinductive activity of various hydrogels.** (a) The typical mandible defect model is a 5-mm diameter round penetrating bone defect in ascending ramus area, and (b, c) the procedure of hydrogel implantation.


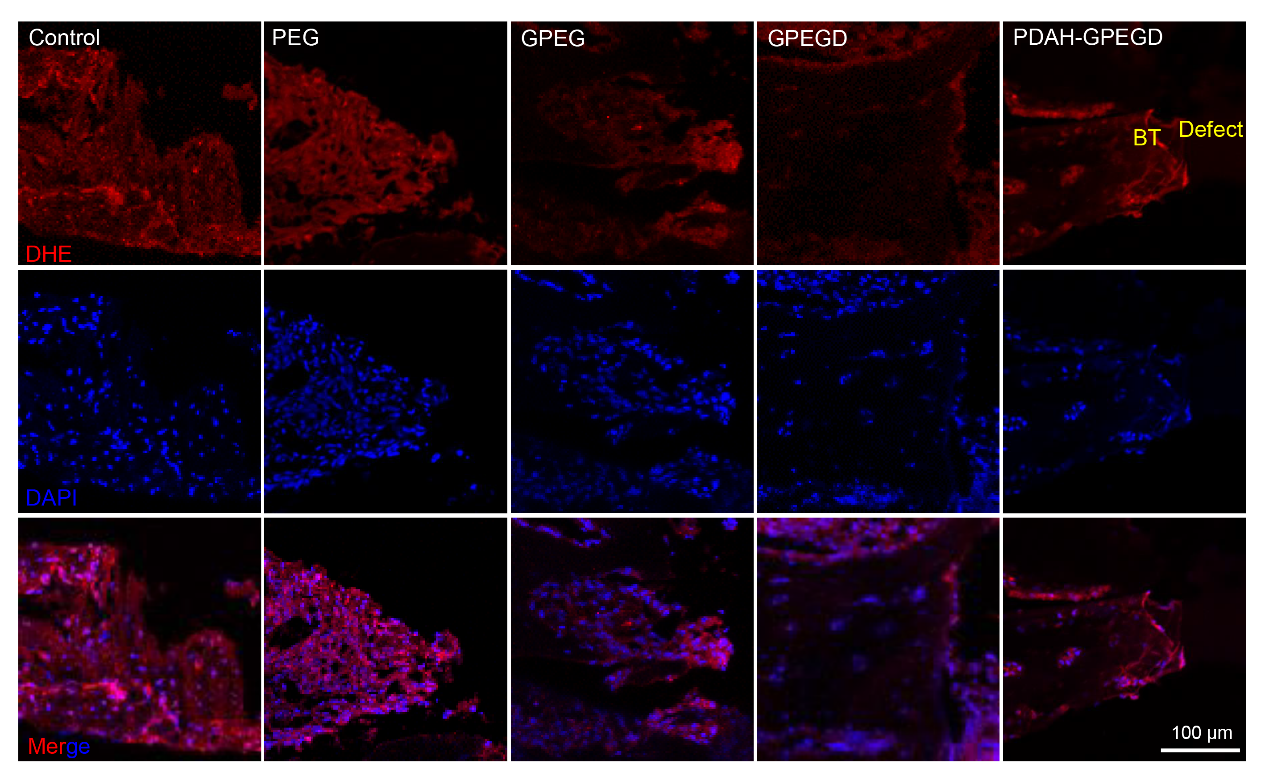


**Figure S9. Antioxidant activity of hydrogels *in vivo*.** Representative DHE staining images of bone tissues (BT) around mandibular bone defects of Sprague-Dawley (SD) rats after treatments for 3 days. The expression of ROS was stained with DHE (red). Nuclei were stained with DAPI (blue).


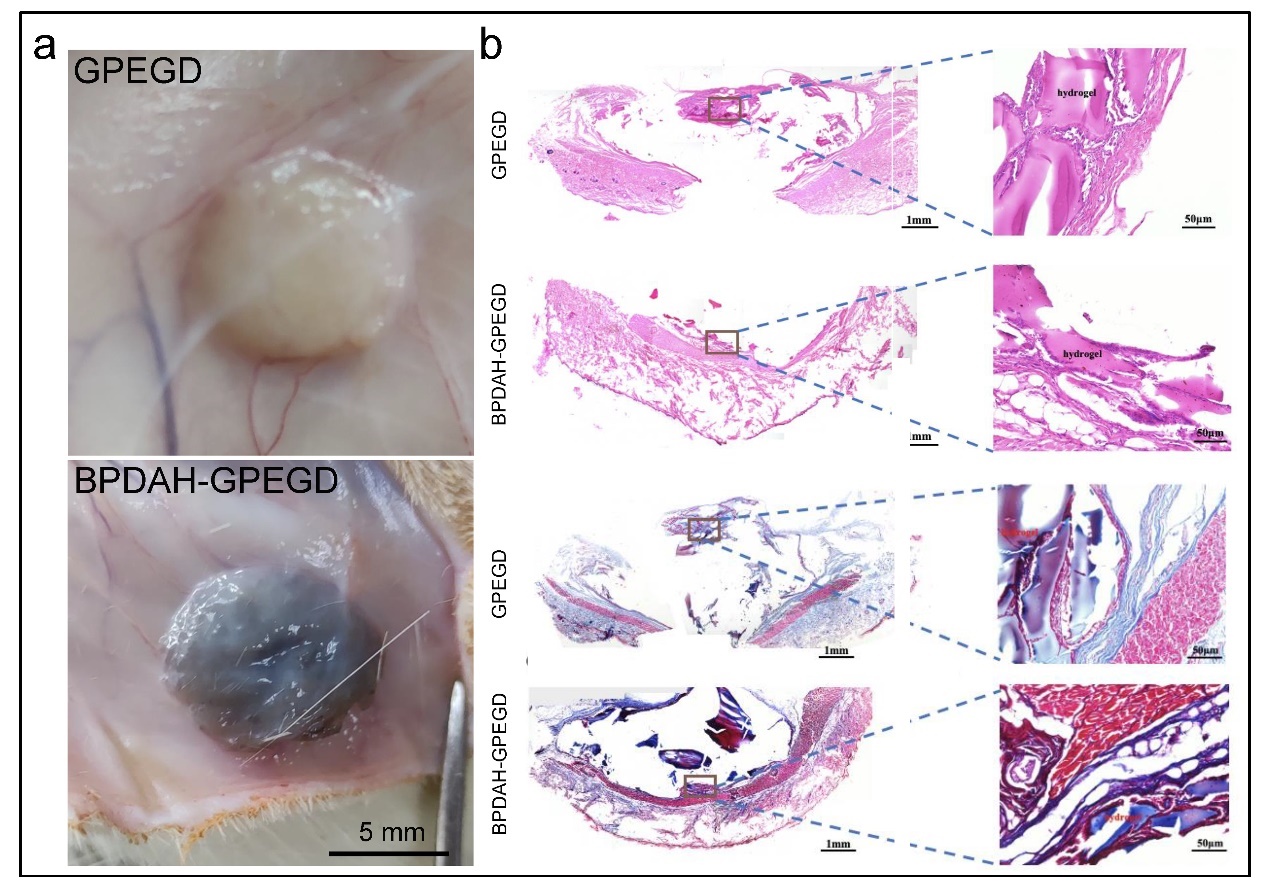


**Figure S10. *In vivo* biocompatibility of GPEGD and BPEGD-GPEGD hydrogels after 2 week-implantation in subdermal implantation of SD rats.** (a) Representative optical images, (b) H&E-stained and Masson’s trichrome-stained histologic sections of hydrogel and surrounding tissues. After 2 weeks post-implantation, H&E and Masson’s trichrome showed that the hydrogels were surrounded by regenerate connective tissue without obvious infiltration of inflammatory cells


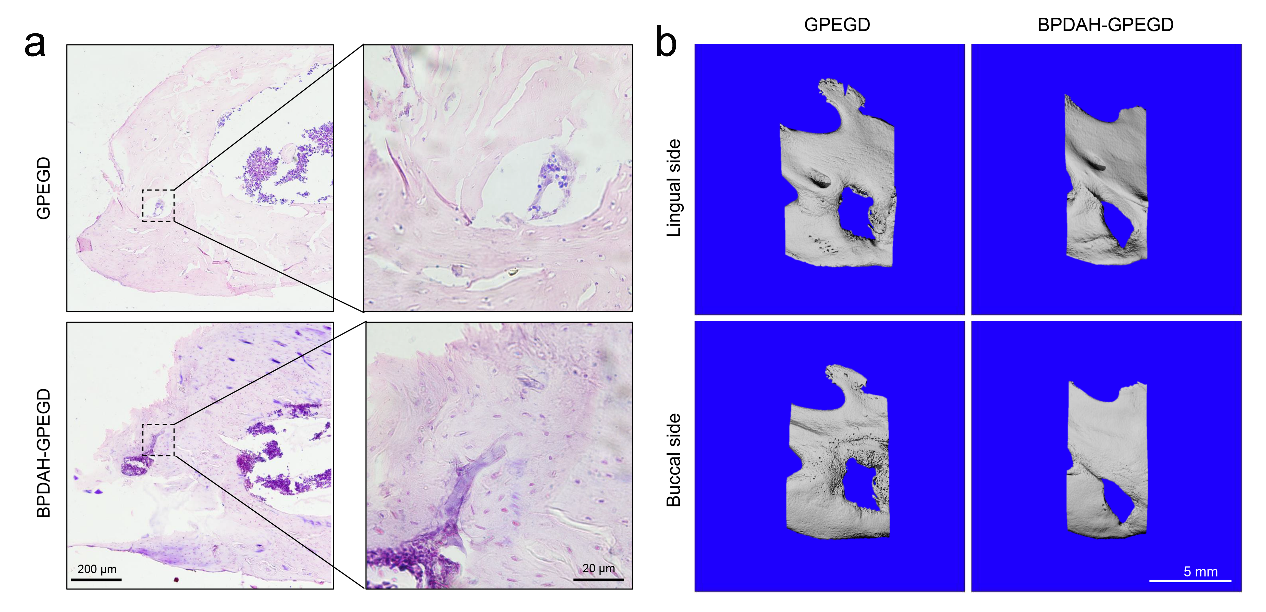


**Figure S11.** (A) Gram-stained histologic sections of mandible decalcified sections after 4 weeks of implantation. (B) Representative Micro-CT images of bone tissue in harvested mandibles obtained from SD rats after treatments for 4 weeks. The Gram staining method was used to detect Gram-positive and Gram-negative bacteria of the hydrogels after the implantation of hydrogels in the mandibular defects for 4 weeks [^8^](#_ENREF_8). Both the Gram-positive and Gram-negative bacteria were not detected in the GPEGD and BPDAH-GPEGD hydrogels. At last, the Micro-CT images viewed from buccal side and lingual side demonstrated that there was no ectopic bone formation in the hydrogels.


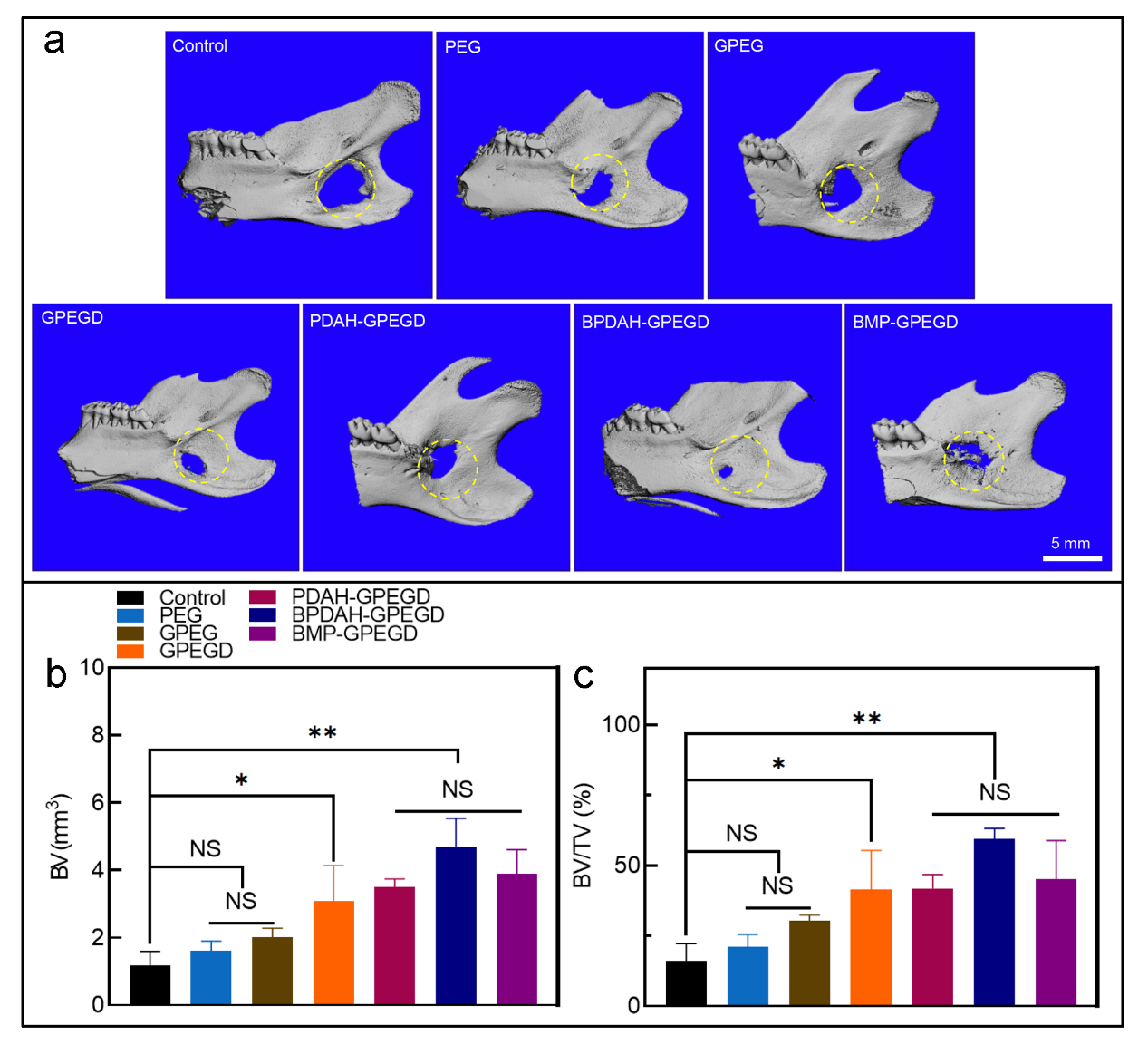


**Figure S12. *In vivo* osteogenesis performance of different hydrogels after 8 week-implantation.** (a) Representative Micro-CT images, and quantitative analysis of (b) the BV and (c) the BV/TV of newly formed bone tissue in harvested mandibles obtained from SD rats after treatments for 8 weeks.


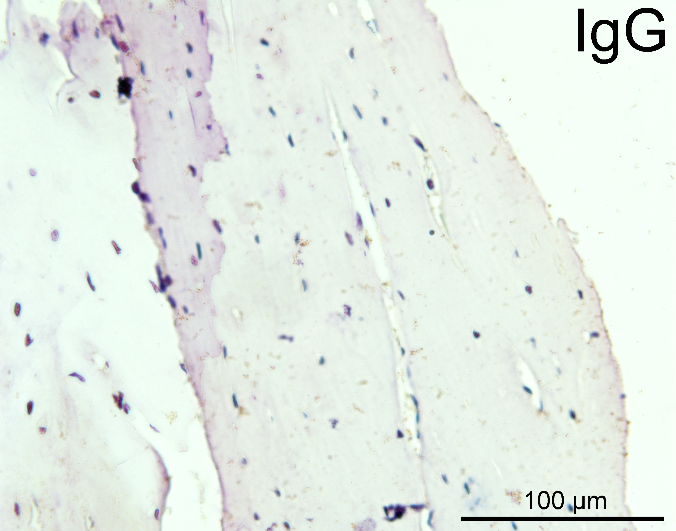


**Figure S13.** Negative control of IHC using IgG to replace specific first antibodies.

**Table S1. The different weights of compositions in various hydrogels.**

| Hydrogels | PEGDA  (mg mL^-1^) | Gelatin  (mg mL^-1^) | DMAEMA  (mg mL^-1^) | BPDAH  (mg mL^-1^) | BMP-2  (μg mL^-1^) |
| --- | --- | --- | --- | --- | --- |
| PEG | 110 | - | - | - | - |
| GPEG | 110 | 100 | - | - | - |
| GPEGD1 | 109 | 100 | 1 | - | - |
| GPEGD3 | 107 | 100 | 3 |  |  |
| GPEGD5 | 105 | 100 | 5 | - | - |
| GPEGD7 | 103 | 100 | 7 | - | - |
| GPEGD9 | 101 | 100 | 9 | - | - |
| GPEGD10 | 100 | 100 | 10 |  | - |
| BPDAH-GPEGD | 110 | 100 | 5 | 1 | - |
| BMP-GPEGD | 110 | 100 | 5 | - | 1 |

**Table S2. Mechanical properties of various hydrogels.**

| Samples | | Compression strain (%) | Compression stress (kPa) | Elastic modulus (kPa) | Toughness  (kJ/m^3^) |
| --- | --- | --- | --- | --- | --- |
| PEG | | 28.28 ± 0.94 | 52.83 ± 10.19 | 89.68 ± 10.19 | 7.04 ± 0.47 |
| GPEG | 44.02 ± 4.13 | | 131.34 ± 10.87 | 80.42 ± 10.88 | 19.69 ± 2.94 |
| GPEGD5 | | 57.36 ± 5.18 | 742.00 ± 19.22 | 179.24 ± 19.22 | 95.73 ± 10.15 |
| PDAH-GPEGD | | 56.85 ± 2.77 | 651.58 ± 15.08 | 158.33 ± 5.08 | 84.98 ± 8.62 |
